# Improved Candidate Drug Mining for Alzheimer's Disease

**DOI:** 10.1155/2014/897653

**Published:** 2014-02-27

**Authors:** Yu-Huei Cheng, Li-Yeh Chuang, Hsueh-Wei Chang, Cheng-Hong Yang

**Affiliations:** ^1^Department of Digital Content Design and Management, Toko University, Chiayi 613, Taiwan; ^2^Department of Chemical Engineering, Institute of Biotechnology and Chemical Engineering, I-Shou University, Kaohsiung 84001, Taiwan; ^3^Department of Biomedical Science and Environmental Biology, Kaohsiung Medical University, Kaohsiung 80708, Taiwan; ^4^Translational Research Center, Kaohsiung Medical University Hospital, Kaohsiung Medical University, Kaohsiung 80708, Taiwan; ^5^Cancer Center, Kaohsiung Medical University Hospital, Kaohsiung Medical University, Kaohsiung 80708, Taiwan; ^6^Institute of Medical Science and Technology, National Sun Yat-Sen University, Kaohsiung 80424, Taiwan; ^7^Department of Electronic Engineering, National Kaohsiung University of Applied Sciences, Kaohsiung 80778, Taiwan

## Abstract

Alzheimer's disease (AD) is the main cause of dementia for older people. Although several antidementia drugs such as donepezil, rivastigmine, galantamine, and memantine have been developed, the effectiveness of AD drug therapy is still far from satisfactory. Recently, the single nucleotide polymorphisms (SNPs) have been chosen as one of the personalized medicine markers. Many pharmacogenomics databases have been developed to provide comprehensive information by associating SNPs with drug responses, disease incidence, and genes that are critical in choosing personalized therapy. However, we found that some information from different sets of pharmacogenomics databases is not sufficient and this may limit the potential functions for pharmacogenomics. To address this problem, we used approximate string matching method and data mining approach to improve the searching of pharmacogenomics database. After computation, we can successfully identify more genes linked to AD and AD-related drugs than previous online searching. These improvements may help to improve the pharmacogenomics of AD for personalized medicine.

## 1. Introduction

Alzheimer's disease (AD), the most common form of dementia, was first reported in 1906 [1]. In 2006, there were about 26.6 million AD patients worldwide and it was also common in southern Taiwan [2]. Although AD has been identified for a long time, most research progress was made in the recent 30 years [3]. However, no definitive cure is available for this disease and eventually it leads to death. Therefore, the drug discovery for Alzheimer's disease remains challenging.

Single nucleotide polymorphisms (SNPs) are the most common variation in human genomes [4]. The importance of SNPs has been reviewed in genome-wide association studies for its association with disease susceptibility and drug metabolism [5, 6]. About 60–90% of the individual variation of drug response depends on pharmacogenomic factors. Therefore, SNP genotyping for candidate genes, pharmacological research, and drug discovery may play an increasingly important role in AD treatment. Meanwhile, increasing amounts of related information require the assistance of bioinformatics to construct the suitable databases and web servers.

Recently, PharmGKB (the Pharmacogenetics and Pharmacogenomics Knowledge Base) has been constructed to provide a comprehensive database for pharmacogenomic studies [7]. PharmGKB provides the pharmacogenetics research network in terms of SNP discovery and drug responses [8] with the fully curated knowledge for drug pathways, drug-related genes, and relationships among genes, drugs, and diseases. However, some information of different functions of PharmGKB is insufficient to allow convenient crosstalking between each other.

To solve this problem, we propose data mining method to improve the searching of pharmacogenomics of AD based on the download dataset of the PharmGKB resource.

## 2. Materials and Methods

**Figure 1:**
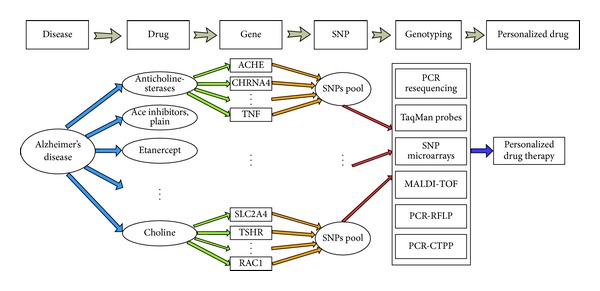
The flowchart for PharmGKB-based pharmacogenomics of AD in this study.

The flowchart for pharmacogenomics in AD for personalized drug studies is shown in Figure 1. First of all, the AD-related drugs and genes are retrieved from PharmGKB download data using approximate string matching method and data mining approach. The genes associated with AD and the genes associated with a single Alzheimer's drug are identified and compared with the online searching of PharmGKB. Then, numerous SNPs of genes associated with AD are identified. Through some SNP genotyping tools or assays, the association studies to AD-related drugs may be evaluated. Finally, the relevant information may be helpful for the personalized drug research.

### 2.1. AD-Related Drugs Using Approximate String Matching Based on PharmGKB Download Data

In order to study the pharmacogenomics of AD, we downloaded the known PharmGKB (the Pharmacogenetics and Pharmacogenomics Knowledge Base) (http://www.pharmgkb.org/downloads/) [9, 10] as source by the approximate string matching method [11] to find out all AD-related drug classes. The meaningful keywords associated with “Alzheimer's disease” are shown in Table 1. Then, these found drug classes are used to find out associated genes by data mining approach. The description of the approximate string matching method for all AD-related drug classes gives a pattern string *P* = *p*_1_ *p*_2_ *p*_3_ ⋯ *p*_*m*_, that is, the meaningful keywords associated with “Alzheimer's disease” and a text string *T* = *t*_1_ *t*_2_ *t*_3_ ⋯ *t*_*n*_, that is, the description for drug and disease retrieved from PharmGKB. Find a substring *T*_*i*,*j*_ = *t*_*i*_ *t*_*i*+1_ *t*_*i*+2_ ⋯ *t*_*j*_ in *T* that has the smallest edit distance [12] to the pattern *P*. The pseudocode for the edit distance is shown in Algorithm 1.

### 2.2. Data Mining Method for PharmGKB Download Data

In this study, we used a priori algorithm [13] for frequent item set mining and association rule learning over PharmGKB. The pseudocode for the a priori algorithm for data mining in PharmGKB is shown in Algorithm 2. At first, a priori algorithm has to find out the frequent gene in drug class for “Alzheimer's disease.” A set of genes can be mined from each drug class. A priori algorithm is a “bottom up” approach, where frequent gene subsets are extended one item at a time (i.e., candidate generation) and groups of candidates are tested against the data. This algorithm is terminated when no further successful extensions are found.

### 2.3. SNP Searching for Genes Using the NCBI dbSNP

Every gene contains numerous SNPs. In order to find out SNPs of single gene for Alzheimer's pharmacogenomics, NCBI dbSNP (http://www.ncbi.nlm.nih.gov/snp) is used to search in the study.

## 3. Results and Discussion

### 3.1. AD Information Based on PharmGKB Search

**Figure 2:**
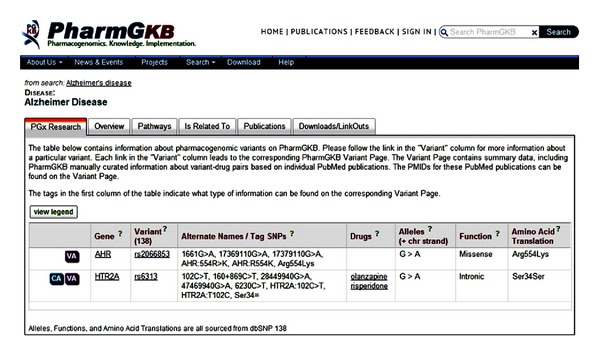
PharmGKB-pharmacogenomics online query for the variant information (SNP rs#ID) of “Alzheimer's disease.” Retrieval source: http://www.pharmgkb.org/disease/PA443319?previousQuery=Alzheimer's%20disease.

**Figure 3:**
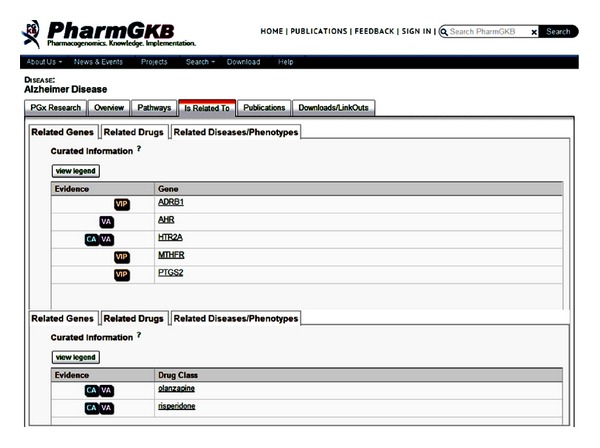
Gene and drug related information of “Alzheimer's disease” online query from PharmGKB. Retrieval source: http://www.pharmgkb.org/disease/PA443319?previousQuery=Alzheimer's%20disease#tabview=table 3&subtab=33.

In PharmGKB online searching, the SNP variants, related genes, and drugs for AD are able to be retrieved. For example, the SNP information such as rs2066853 and rs6313 is provided (Figure 2). As shown in Figure 3, the AD-related genes such as ADRB1, AHR, HTR2A, MTHFR, and PTGS2 are identified and the related drugs such as olanzapine and risperidone are searched. This information may assist the researchers to study the pharmacogenomics of AD. Unfortunately, this PharmGKB online searching just provides limited information and it insufficiently copes with the complexity of the drug researches for Alzheimer's personalized medicine.

### 3.2. PharmGKB-Based Data Mining of AD Information of Drug Classes or Gene Symbols

In current study, our proposed method is used to perform data mining for PharmGKB download data in terms of the keyword “Alzheimer's disease.” As shown in Table 2, 22 kinds of AD-related drug classes are identified from “drug classes” of PharmGKB. Their corresponding PharmGKB accession ID, PubMed PMID, and the number of genes that are associated with AD-related drug classes are also presented. In total, 495 genes are identified for AD information of drug classes (see Supplementary file 1: gene information includes PharmGKB Accession Id, gene symbol, and publications are providing in different classes; it is available online at http://dx.doi.org/10.1155/2014/897653). Alternatively, 99 genes associated with AD are identified from “gene symbols” of PharmGKB in terms of the keyword “Alzheimer's disease.” These results suggest that the same keyword, for example, Alzheimer's disease, may identify different numbers of AD-associated genes between “drug classes” or “gene symbols” of PharmGKB.

After detailed examination, 67 genes in the gene symbols searching (bold fonts of gene names as shown in Table 3) are absent from the genes in the drug class searching (Table 2). Furthermore, genes corresponding to the drug “memantine” listed in Table 2 (drug classes) are not found in Table 3 (gene symbols). Therefore, some current drugs have identified a small number of AD-related genes in the drug class searching; however, the remaining AD-related genes that may affect AD-related drugs may be partly discovered in the gene symbols searching. These novelly identified AD-related genes may be the potential candidates for further drug development of AD. These results demonstrated that our proposed data mining method may be an improved AD pharmacogenomics study.

### 3.3. SNP Information of AD-Related Genes

The SNP statuses for 99 AD-related genes are also provided in Table 3. This SNP status for each gene is calculated from the online NCBI dbSNP queries. In general, many SNPs are found in these AD-related genes. Some SNPs of these genes have been reported to be associated with AD. For example, the APOE gene is found in Table 3 and one of its SNPs, such as ApoE epsilon 4 allele, has been reported to be associated with AD [14]. With suitable tools for SNP genotyping, these SNP candidates are warranted for the pharmacogenomics research of AD.

Currently, there are many high throughput SNP genotyping methods developed (as shown in Figure 1), including PCR resequencing [15], TaqMan probes [16], SNP microarrays [17], Matrix Assisted Laser Desorption/Ionization-Time of Flight (MALDI-TOF) [18], and others [19, 20]. Furthermore, some SNP genotyping tools or databases are also developed, such as SNP-RFLPing2 for comprehensive PCR-RFLP information based on SNPs [21–24], algorithmic PCR-RFLP primer design and restriction enzymes for SNP genotyping [25, 26], and primer design for PCR-confronting two-pair primers (PCR-CTPP) [27, 28]. These tools and methods can provide useful and convenient information for SNP genotyping in the AD pharmacogenomics studies.

## 4. Conclusions

AD is the most common form of dementia for older people. The pharmacogenomics of AD still remains a challenge. In this study, we propose the pharmGKB-based data mining method to improve the gene discoveries for the potential AD-related drug candidates. With the assistance of bioinformatics, this improvement can help researchers to develop personal therapeutic drugs of AD.

## Supplementary Material

Gene information includes PharmGKB Accession Id, gene symbol, and publications are providing in different classes.

## Figures and Tables

**Algorithm 1:**
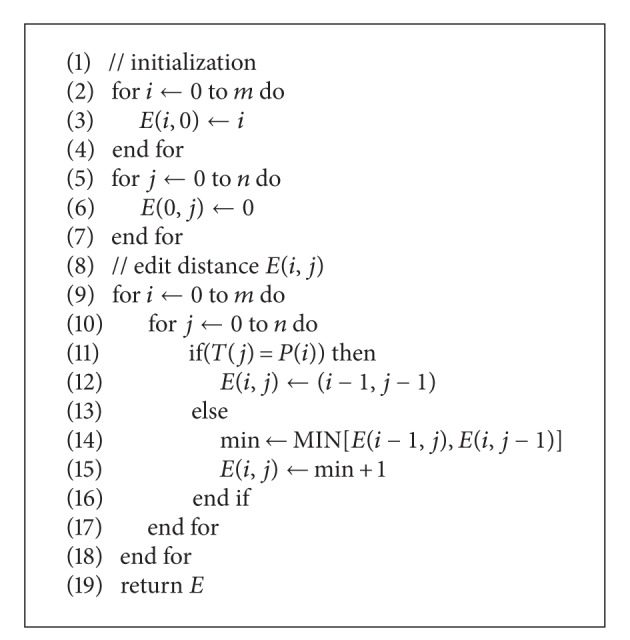
Pseudocode for the edit distance used for approximate string matching.

**Algorithm 2:**
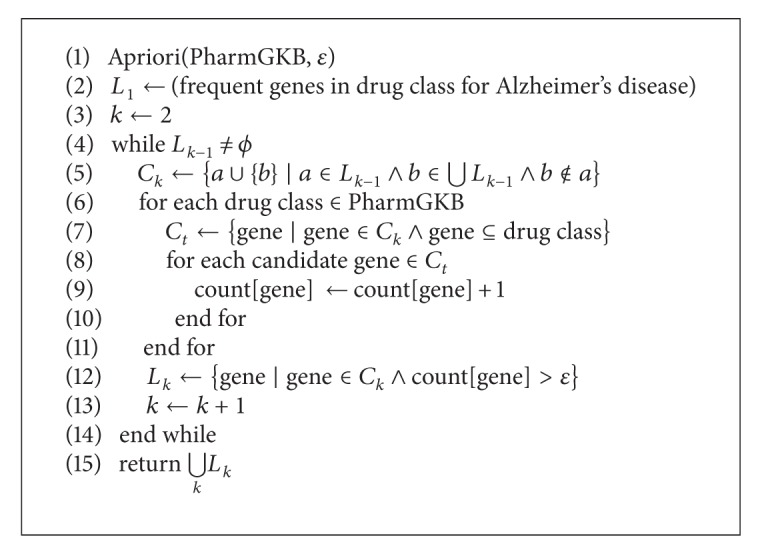
Pseudocode for a priori algorithm for the data mining in PharmGKB, where ε is a support threshold, *L* is the frequent gene subsets that satisfy the support threshold, *k* is the number of current iterations, and *C* is the candidate set, and count[gene] accesses a field of the data structure that represents gene candidate set.

**Table 1:** The meaningful keywords associated with “Alzheimer's disease” are retrieved from PharmGKB and they are applied to discover the drug classes*.

ID	Keywords
1	AD
2	Alzheimer's disease
3	AD—Alzheimer's disease
4	Acute Confusional Senile Dementia
5	Alzheimer Dementia, Presenile
6	Alzheimer Disease, Early Onset
7	Alzheimer Disease, Late Onset
8	Alzheimer Type Dementia
9	Alzheimer Type Senile Dementia
10	Alzheimer's Disease, Focal Onset
11	Alzheimer's disease, NOS
12	Dementia, Alzheimer Type
13	Dementia, Presenile
14	Dementia, Presenile Alzheimer
15	Dementia, Primary Senile Degenerative
16	Dementia, Senile
17	Dementias, Presenile
18	Dementias, Senile
19	Disease, Alzheimer
20	Disease, Alzheimer's
21	Early Onset Alzheimer Disease
22	Focal Onset Alzheimer's Disease
23	Late Onset Alzheimer Disease
24	Presenile Alzheimer Dementia
25	Presenile Dementia
26	Presenile Dementias
27	Primary Senile Degerative Dementia
28	Senile Dementia
29	Senile Dementia, Acute Confusional
30	Senile Dementia, Alzheimer Type
31	Senile Dementias
32	MeSH: D000544 (Alzheimer Disease)
33	MedDRA: 10001896 (Alzheimer's disease)
34	NDFRT: N0000000363 (Alzheimer Disease [Disease/Finding])
35	SnoMedCT: 26929004 (Alzheimer's disease)
36	UMLS: C0002395 (C0002395)

*Drug class is one of the functions listed in the ParamGKB download data.

**Table 2:** PharmGKB-based data mining results in terms of the PharmGKB accession ID, drug class, publications, and the number of gene information of Alzheimer's disease.

No.	PharmGKB accession ID	Drug classes	Publications^∗1^	Gene no.^∗2^
1	PA164712423	Anticholinesterases	PMID: 20644562 20644562 14674789	6
2	PA164712308	Ace inhibitors, plain	PMID: 17362841	24
3	PA449515	Etanercept	PMID: 19027875	12
4	PA451262	Rivastigmine	PMID: 20644562 16323253 17082448 20644562 15289797 17522596	2
5	PA450243	Lithium	PMID: 17082448	13
6	PA10384	Anti-inflammatory and antirheumatic products, nonsteroids	PMID: 17082448 17082448	11
7	PA449760	Glatiramer acetate	PMID: 17082448	4
8	PA133950441	Hmg coa reductase inhibitors	PMID: 17082448	39
9	PA151958596	Curcumin	PMID: 17082448	2
10	PA451898	Vitamin c	PMID: 17082448	16
11	PA451900	Vitamin e	PMID: 17082448	1
12	PA452229	Antidepressants	PMID: 17082448	43
13	PA452233	Antipsychotics	PMID: 17082448	46
14	PA449726	Galantamine	PMID: 20644562 16323253 17082448 15853556 20644562 14674789 12177686	7
15	PA10364	Memantine	PMID: 17082448	0
16	PA451283	Rosiglitazone	PMID: 16770341	34
17	PA448031	Acetylcholine	PMID: 15695160	8
18	PA450626	Nicotine	PMID: 15695160	88
19	PA137179528	Nimesulide	PMID: 16331303 11810182	3
20	PA449394	Donepezil	PMID: 20859244 20644562 16323253 16424819 17082448 20644562 1973817012142731	9
21	PA451576	Tacrine	PMID: 9521254 17082448 10801254 9777427 18004213	6
22	PA448976	Choline	PMID: 8618881	122

^∗1^PMID: PubMed article ID number.

^∗2^The full gene names for each of the “drug classes” have been provided in the Supplementary file 1.

**Table 3:** PharmGKB-based data mining results of gene symbols of Alzheimer's disease and NCBI dbSNP-based query results for SNP number for the genes of Alzheimer's disease.

No.	PharmGKB accession ID	Gene symbols*	SNP no.	No.	PharmGKB accession ID	Gene symbols*	SNP no.	No.	PharmGKB accession ID	Gene symbols*	SNP no.
1	PA20	ACHE	899	34	PA37597	**ZNF225**	813	67	PA125	CYP2C8	993
2	PA26490	CHRNA4	1518	35	PA38499	**DEFB123**	330	68	PA126	CYP2C9	1605
3	PA128	CYP2D6	482	36	PA134902026	**SORCS2**	19073	69	PA30864	MME	3323
4	PA130	CYP3A4	899	37	PA134949387	**SORCS3**	13969	70	PA142671271	**NCSTN**	741
5	PA26620	**CLU**	644	38	PA38274	**TOMM40**	462	71	PA36153	**SST**	120
6	PA26855	**CR1**	19859	39	PA162397694	**NLRC5**	2297	72	PA36457	**TF**	1501
7	PA33287	**PICALM**	3169	40	PA24641	**AHR**	991	73	PA31930	OPCML	28437
8	PA46	**ALOX5**	1992	41	PA134950706	**DNMBP**	3312	74	PA29561	**HTR7**	2623
9	PA293	PTGS2	579	42	PA24910	APP	9411	75	PA162393285	**KIF20B**	2109
10	PA108	CETP	1246	43	PA238	MAPT	4399	76	PA26971	**CSRP3**	907
11	PA32996	**PCDH11X**	15199	44	PA128394579	TMED10	1079	77	PA231	**LMNA**	1486
12	PA24507	**ADAM12**	10827	45	PA162397475	**NGF**	1286	78	PA27029	**CTSD**	460
13	PA25165	**ATP8A1**	5983	46	PA25232	**BACE1**	794	79	PA29629	**IDE**	2755
14	PA26243	**CD86**	1385	47	PA36022	**SORL1**	4394	80	PA31374	**MYH7**	1157
15	PA26935	**CSF1**	569	48	PA33796	**PRNP**	452	81	PA272	**PLN**	343
16	PA27342	**DISC1**	11813	49	PA37302	**VEGFA**	561	82	PA33855	**PSEN1**	2343
17	PA28597	**GBP2**	625	50	PA114	CHRNA7	3714	83	PA33856	**PSEN2**	959
18	PA220	**KCNMA1**	19081	51	PA37155	**UBQLN1**	1400	84	PA304	SCN5A	3380
19	PA25512	**KCTD12**	235	52	PA26123	**CBS**	924	85	PA36638	TNNT2	739
20	PA164724093	NOS2	1820	53	PA26976	**CST3**	233	86	PA139	ACE	1108
21	PA33614	**PPP1R11**	215	54	PA25623	C1QB	356	87	PA37935	**SIRT1**	1145
22	PA143485670	**WWC1**	5070	55	PA162380954	**CALHM1**	247	88	PA55	APOE	184
23	PA37596	**ZNF224**	490	56	PA30748	**MEOX2**	2140	89	PA24357	**A2M**	1385
24	PA162380963	**CALHM2**	192	57	PA26448	CHAT	2572	90	PA192	HTR1A	186
25	PA51	APOC1	243	58	PA38239	**CLSTN2**	15608	91	PA182	GSTM1	264
26	PA34958	**ATXN1**	11910	59	PA134952303	**NMNAT3**	39	92	PA183	GSTT1	200
27	PA26210	**CD33**	465	60	PA134904440	**C1orf49**	348	93	PA268	ABCB4	1915
28	PA28478	**GAB2**	5119	61	PA134864387	**RALGPS2**	3980	94	PA115	CHRNB2	698
29	PA34052	**PVRL2**	1344	62	PA134870196	**RGSL1**	3300	95	PA156	**ESR1**	10108
30	PA37754	**ZNRD1**	316	63	PA25294	BCHE	1796	96	PA134934259	**GAPDHS**	361
31	PA38114	**TRIM15**	466	64	PA120	CRP	977	97	PA245	MTHFR	790
32	PA134927803	**MTHFD1L**	7229	65	PA127	**CYP2C18**	1353	98	PA36458	**TFAM**	376
33	PA144596420	**INTS1**	1820	66	PA124	CYP2C19	2692	99	PA435	TNF	268

*Gene names in bold fonts are not identified in Table 2.
