# Supplementary material for: Improved Candidate Drug Mining for Alzheimer's Disease
Source: Biomed Res Int. 2014 Feb 27;2014:897653. doi: 10.1155/2014/897653 (PMC3955684; doi:10.1155/2014/897653)
Supplement: Supplementary file 1 — Gene information includes PharmGKB Accession Id, gene symbol, and publications are providing in different classes. [file 897653.f1.doc]

**Supplementary file 1**

The following tables show the gene information in different drug class. The shown information includes PharmGKB Accession Id, gene symbol, and publications.

Table S1. The gene information for “Anticholinesterases”

| **ID** | **PharmGKB Accession Id** | **Gene Symbol** | **Publications** |
| --- | --- | --- | --- |
|  | [PA20](http://www.pharmgkb.org/do/serve?objId=PA20&objCls=Gene) | ACHE | PMID: [14674789](http://www.ncbi.nlm.nih.gov/pubmed/14674789) |
|  | [PA26490](http://www.pharmgkb.org/do/serve?objId=PA26490&objCls=Gene) | CHRNA4 | PMID: [14674789](http://www.ncbi.nlm.nih.gov/pubmed/14674789) |
|  | [PA128](http://www.pharmgkb.org/do/serve?objId=PA128&objCls=Gene) | CYP2D6 | PMID: [14674789](http://www.ncbi.nlm.nih.gov/pubmed/14674789) |
|  | [PA130](http://www.pharmgkb.org/do/serve?objId=PA130&objCls=Gene) | CYP3A4 | PMID: [14674789](http://www.ncbi.nlm.nih.gov/pubmed/14674789) |
|  | [PA55](http://www.pharmgkb.org/do/serve?objId=PA55&objCls=Gene) | APOE | PMID: [20644562](http://www.ncbi.nlm.nih.gov/pubmed/20644562) |
|  | [PA25294](http://www.pharmgkb.org/do/serve?objId=PA25294&objCls=Gene) | BCHE | PMID: [20644562](http://www.ncbi.nlm.nih.gov/pubmed/20644562) |

Table S2. The gene information for “Ace Inhibitors, Plain”

| **ID** | **PharmGKB Accession Id** | **Gene Symbol** | **Publications** |
| --- | --- | --- | --- |
|  | [PA425](http://www.pharmgkb.org/do/serve?objId=PA425&objCls=Gene) | ACE2 | PMID: [18449520](http://www.ncbi.nlm.nih.gov/pubmed/18449520) |
|  | [PA42](http://www.pharmgkb.org/do/serve?objId=PA42&objCls=Gene) | AGT | PMID: [20712529](http://www.ncbi.nlm.nih.gov/pubmed/20712529) [19752885](http://www.ncbi.nlm.nih.gov/pubmed/19752885) [15174896](http://www.ncbi.nlm.nih.gov/pubmed/15174896) [19082699](http://www.ncbi.nlm.nih.gov/pubmed/19082699) [17376010](http://www.ncbi.nlm.nih.gov/pubmed/17376010) [14643574](http://www.ncbi.nlm.nih.gov/pubmed/14643574) [8903618](http://www.ncbi.nlm.nih.gov/pubmed/8903618) [17299437](http://www.ncbi.nlm.nih.gov/pubmed/17299437) |
|  | [PA43](http://www.pharmgkb.org/do/serve?objId=PA43&objCls=Gene) | AGTR1 | PMID: [20712529](http://www.ncbi.nlm.nih.gov/pubmed/20712529) [12522467](http://www.ncbi.nlm.nih.gov/pubmed/12522467) [19752885](http://www.ncbi.nlm.nih.gov/pubmed/19752885) [15174896](http://www.ncbi.nlm.nih.gov/pubmed/15174896) [19082699](http://www.ncbi.nlm.nih.gov/pubmed/19082699) [17376010](http://www.ncbi.nlm.nih.gov/pubmed/17376010) [8903618](http://www.ncbi.nlm.nih.gov/pubmed/8903618) [18449520](http://www.ncbi.nlm.nih.gov/pubmed/18449520) [18347611](http://www.ncbi.nlm.nih.gov/pubmed/18347611) [20712529](http://www.ncbi.nlm.nih.gov/pubmed/20712529) |
|  | [PA44](http://www.pharmgkb.org/do/serve?objId=PA44&objCls=Gene) | AGTR2 | PMID: [20712529](http://www.ncbi.nlm.nih.gov/pubmed/20712529) [19082699](http://www.ncbi.nlm.nih.gov/pubmed/19082699) [17376010](http://www.ncbi.nlm.nih.gov/pubmed/17376010) [8903618](http://www.ncbi.nlm.nih.gov/pubmed/8903618) |
|  | [PA26623](http://www.pharmgkb.org/do/serve?objId=PA26623&objCls=Gene) | CMA1 | PMID: [9535416](http://www.ncbi.nlm.nih.gov/pubmed/9535416) |
|  | [PA225](http://www.pharmgkb.org/do/serve?objId=PA225&objCls=Gene) | KNG1 | PMID: [20712529](http://www.ncbi.nlm.nih.gov/pubmed/20712529) [19082699](http://www.ncbi.nlm.nih.gov/pubmed/19082699) [9207629](http://www.ncbi.nlm.nih.gov/pubmed/9207629) [12235256](http://www.ncbi.nlm.nih.gov/pubmed/12235256) |
|  | [PA30643](http://www.pharmgkb.org/do/serve?objId=PA30643&objCls=Gene) | MAS1 | PMID: [18449520](http://www.ncbi.nlm.nih.gov/pubmed/18449520) |
|  | [PA254](http://www.pharmgkb.org/do/serve?objId=PA254&objCls=Gene) | NOS3 | PMID: [20712529](http://www.ncbi.nlm.nih.gov/pubmed/20712529) [19082699](http://www.ncbi.nlm.nih.gov/pubmed/19082699) [12785010](http://www.ncbi.nlm.nih.gov/pubmed/12785010) [9207629](http://www.ncbi.nlm.nih.gov/pubmed/9207629) |
|  | [PA297](http://www.pharmgkb.org/do/serve?objId=PA297&objCls=Gene) | REN | PMID: [20712529](http://www.ncbi.nlm.nih.gov/pubmed/20712529) [19082699](http://www.ncbi.nlm.nih.gov/pubmed/19082699) [17376010](http://www.ncbi.nlm.nih.gov/pubmed/17376010) |
|  | [PA80](http://www.pharmgkb.org/do/serve?objId=PA80&objCls=Gene) | BDKRB2 | PMID: [20712529](http://www.ncbi.nlm.nih.gov/pubmed/20712529) [12676166](http://www.ncbi.nlm.nih.gov/pubmed/12676166) [19082699](http://www.ncbi.nlm.nih.gov/pubmed/19082699) [9293967](http://www.ncbi.nlm.nih.gov/pubmed/9293967) [21052031](http://www.ncbi.nlm.nih.gov/pubmed/21052031) [9535416](http://www.ncbi.nlm.nih.gov/pubmed/9535416) [10904024](http://www.ncbi.nlm.nih.gov/pubmed/10904024) [11699055](http://www.ncbi.nlm.nih.gov/pubmed/11699055) [12522467](http://www.ncbi.nlm.nih.gov/pubmed/12522467) |
|  | [PA31734](http://www.pharmgkb.org/do/serve?objId=PA31734&objCls=Gene) | NPPB | PMID: [10335726](http://www.ncbi.nlm.nih.gov/pubmed/10335726) |
|  | [PA24910](http://www.pharmgkb.org/do/serve?objId=PA24910&objCls=Gene) | APP | PMID: [17362841](http://www.ncbi.nlm.nih.gov/pubmed/17362841) |
|  | [PA24415](http://www.pharmgkb.org/do/serve?objId=PA24415&objCls=Gene) | ABO | PMID: [20066004](http://www.ncbi.nlm.nih.gov/pubmed/20066004) |
|  | [PA245](http://www.pharmgkb.org/do/serve?objId=PA245&objCls=Gene) | MTHFR | PMID: [16081343](http://www.ncbi.nlm.nih.gov/pubmed/16081343) |
|  | [PA26087](http://www.pharmgkb.org/do/serve?objId=PA26087&objCls=Gene) | CASP3 | PMID: [12785010](http://www.ncbi.nlm.nih.gov/pubmed/12785010) |
|  | [PA164724093](http://www.pharmgkb.org/do/serve?objId=PA164724093&objCls=Gene) | NOS2 | PMID: [12785010](http://www.ncbi.nlm.nih.gov/pubmed/12785010) |
|  | [PA79](http://www.pharmgkb.org/do/serve?objId=PA79&objCls=Gene) | BDKRB1 | PMID: [20712529](http://www.ncbi.nlm.nih.gov/pubmed/20712529) [12676166](http://www.ncbi.nlm.nih.gov/pubmed/12676166) [19082699](http://www.ncbi.nlm.nih.gov/pubmed/19082699) [20712529](http://www.ncbi.nlm.nih.gov/pubmed/20712529) |
|  | [PA133](http://www.pharmgkb.org/do/serve?objId=PA133&objCls=Gene) | CYP11B1 | PMID: [20712529](http://www.ncbi.nlm.nih.gov/pubmed/20712529) [19082699](http://www.ncbi.nlm.nih.gov/pubmed/19082699) |
|  | [PA134](http://www.pharmgkb.org/do/serve?objId=PA134&objCls=Gene) | CYP11B2 | PMID: [20712529](http://www.ncbi.nlm.nih.gov/pubmed/20712529) [19082699](http://www.ncbi.nlm.nih.gov/pubmed/19082699) |
|  | [PA224](http://www.pharmgkb.org/do/serve?objId=PA224&objCls=Gene) | KLK1 | PMID :[20712529](http://www.ncbi.nlm.nih.gov/pubmed/20712529) [19082699](http://www.ncbi.nlm.nih.gov/pubmed/19082699) |
|  | [PA288](http://www.pharmgkb.org/do/serve?objId=PA288&objCls=Gene) | PTGER3 | PMID: [21052031](http://www.ncbi.nlm.nih.gov/pubmed/21052031) |
|  | [PA30864](http://www.pharmgkb.org/do/serve?objId=PA30864&objCls=Gene) | MME | PMID: [21052031](http://www.ncbi.nlm.nih.gov/pubmed/21052031) |
|  | [PA139](http://www.pharmgkb.org/do/serve?objId=PA139&objCls=Gene) | ACE | PMID: [15174896](http://www.ncbi.nlm.nih.gov/pubmed/15174896) [17376010](http://www.ncbi.nlm.nih.gov/pubmed/17376010) [9293967](http://www.ncbi.nlm.nih.gov/pubmed/9293967) [10335726](http://www.ncbi.nlm.nih.gov/pubmed/10335726) [12235256](http://www.ncbi.nlm.nih.gov/pubmed/12235256) [16242049](http://www.ncbi.nlm.nih.gov/pubmed/16242049) [21052031](http://www.ncbi.nlm.nih.gov/pubmed/21052031) [12522467](http://www.ncbi.nlm.nih.gov/pubmed/12522467) [19198819](http://www.ncbi.nlm.nih.gov/pubmed/19198819) [17362841](http://www.ncbi.nlm.nih.gov/pubmed/17362841) [11699055](http://www.ncbi.nlm.nih.gov/pubmed/11699055) [19082699](http://www.ncbi.nlm.nih.gov/pubmed/19082699) [8903618](http://www.ncbi.nlm.nih.gov/pubmed/8903618) [19696696](http://www.ncbi.nlm.nih.gov/pubmed/19696696) [19497121](http://www.ncbi.nlm.nih.gov/pubmed/19497121) [18449520](http://www.ncbi.nlm.nih.gov/pubmed/18449520) [9690230](http://www.ncbi.nlm.nih.gov/pubmed/9690230) [19752885](http://www.ncbi.nlm.nih.gov/pubmed/19752885) [15861031](http://www.ncbi.nlm.nih.gov/pubmed/15861031) [16689556](http://www.ncbi.nlm.nih.gov/pubmed/16689556) [10620209](http://www.ncbi.nlm.nih.gov/pubmed/10620209) [19898265](http://www.ncbi.nlm.nih.gov/pubmed/19898265) [9535416](http://www.ncbi.nlm.nih.gov/pubmed/9535416) [20712529](http://www.ncbi.nlm.nih.gov/pubmed/20712529) [20066004](http://www.ncbi.nlm.nih.gov/pubmed/20066004) [9207629](http://www.ncbi.nlm.nih.gov/pubmed/9207629) [17033615](http://www.ncbi.nlm.nih.gov/pubmed/17033615) [18520711](http://www.ncbi.nlm.nih.gov/pubmed/18520711) [16679330](http://www.ncbi.nlm.nih.gov/pubmed/16679330) |
|  | [PA34742](http://www.pharmgkb.org/do/serve?objId=PA34742&objCls=Gene) | RPL38P1 | PMID: none |

Table S3. The gene information for “etanercept”

| **ID** | **PharmGKB Accession Id** | **Gene Symbol** | **Publications** |
| --- | --- | --- | --- |
|  | [PA36605](http://www.pharmgkb.org/do/serve?objId=PA36605&objCls=Gene) | TNFRSF14 | PMID: [19837186](http://www.ncbi.nlm.nih.gov/pubmed/19837186) |
|  | [PA36609](http://www.pharmgkb.org/do/serve?objId=PA36609&objCls=Gene) | TNFRSF1A | PMID: [19837186](http://www.ncbi.nlm.nih.gov/pubmed/19837186) |
|  | [PA36610](http://www.pharmgkb.org/do/serve?objId=PA36610&objCls=Gene) | TNFRSF1B | PMID: [19837186](http://www.ncbi.nlm.nih.gov/pubmed/19837186) |
|  | [PA36085](http://www.pharmgkb.org/do/serve?objId=PA36085&objCls=Gene) | SPP1 | PMID: [19684155](http://www.ncbi.nlm.nih.gov/pubmed/19684155) |
|  | [PA134964409](http://www.pharmgkb.org/do/serve?objId=PA134964409&objCls=Gene) | GBP6 | PMID: none |
|  | [PA134925480](http://www.pharmgkb.org/do/serve?objId=PA134925480&objCls=Gene) | LASS6 | PMID: none |
|  | [PA33529](http://www.pharmgkb.org/do/serve?objId=PA33529&objCls=Gene) | PON1 | PMID: none |
|  | [PA33661](http://www.pharmgkb.org/do/serve?objId=PA33661&objCls=Gene) | PPP1R9A | PMID: none |
|  | [PA134886513](http://www.pharmgkb.org/do/serve?objId=PA134886513&objCls=Gene) | MOBKL2B | PMID: none |
|  | [PA134908144](http://www.pharmgkb.org/do/serve?objId=PA134908144&objCls=Gene) | C9orf72 | PMID: none |
|  | [PA435](http://www.pharmgkb.org/do/serve?objId=PA435&objCls=Gene) | TNF | PMID: [17638513](http://www.ncbi.nlm.nih.gov/pubmed/17638513)[1](http://www.ncbi.nlm.nih.gov/pubmed/17275732) 7275732 |
|  | [PA30474](http://www.pharmgkb.org/do/serve?objId=PA30474&objCls=Gene) | LTA | PMID: [15457442](http://www.ncbi.nlm.nih.gov/pubmed/15457442) [19837186](http://www.ncbi.nlm.nih.gov/pubmed/19837186) |

Table S4. The gene information for “rivastigmine”

| **ID** | **PharmGKB Accession Id** | **Gene Symbol** | **Publications** |
| --- | --- | --- | --- |
|  | [PA25294](http://www.pharmgkb.org/do/serve?objId=PA25294&objCls=Gene) | BCHE | PMID: [20644562](http://www.ncbi.nlm.nih.gov/pubmed/20644562) [19617863](http://www.ncbi.nlm.nih.gov/pubmed/19617863) [17522596](http://www.ncbi.nlm.nih.gov/pubmed/17522596) [12668920](http://www.ncbi.nlm.nih.gov/pubmed/12668920) |
|  | [PA55](http://www.pharmgkb.org/do/serve?objId=PA55&objCls=Gene) | APOE | PMID: [20644562](http://www.ncbi.nlm.nih.gov/pubmed/20644562) [15289797](http://www.ncbi.nlm.nih.gov/pubmed/15289797) |

Table S5. The gene information for “lithium”

| **ID** | **PharmGKB Accession Id** | **Gene Symbol** | **Publications** |
| --- | --- | --- | --- |
|  | [PA25335](http://www.pharmgkb.org/do/serve?objId=PA25335&objCls=Gene) | BDNF | PMID: [19018715](http://www.ncbi.nlm.nih.gov/pubmed/19018715) [20453536](http://www.ncbi.nlm.nih.gov/pubmed/20453536) |
|  | [PA31818](http://www.pharmgkb.org/do/serve?objId=PA31818&objCls=Gene) | NTRK2 | PMID: [19018715](http://www.ncbi.nlm.nih.gov/pubmed/19018715) |
|  | [PA26016](http://www.pharmgkb.org/do/serve?objId=PA26016&objCls=Gene) | CACNG2 | PMID: [18408563](http://www.ncbi.nlm.nih.gov/pubmed/18408563) |
|  | [PA238](http://www.pharmgkb.org/do/serve?objId=PA238&objCls=Gene) | MAPT | PMID: [17082448](http://www.ncbi.nlm.nih.gov/pubmed/17082448) |
|  | [PA30074](http://www.pharmgkb.org/do/serve?objId=PA30074&objCls=Gene) | KCNQ2 | PMID: [16733521](http://www.ncbi.nlm.nih.gov/pubmed/16733521) |
|  | [PA33670](http://www.pharmgkb.org/do/serve?objId=PA33670&objCls=Gene) | PPP2R2C | PMID: [16733521](http://www.ncbi.nlm.nih.gov/pubmed/16733521) |
|  | [PA29009](http://www.pharmgkb.org/do/serve?objId=PA29009&objCls=Gene) | GSK3B | PMID: [16484495](http://www.ncbi.nlm.nih.gov/pubmed/16484495) [20453536](http://www.ncbi.nlm.nih.gov/pubmed/20453536) [20102306](http://www.ncbi.nlm.nih.gov/pubmed/20102306) |
|  | [PA31748](http://www.pharmgkb.org/do/serve?objId=PA31748&objCls=Gene) | NR1D1 | PMID: [16484495](http://www.ncbi.nlm.nih.gov/pubmed/16484495) |
|  | [PA29880](http://www.pharmgkb.org/do/serve?objId=PA29880&objCls=Gene) | INPP1 | PMID: [9682271](http://www.ncbi.nlm.nih.gov/pubmed/9682271) |
|  | [PA312](http://www.pharmgkb.org/do/serve?objId=PA312&objCls=Gene) | SLC6A4 | PMID: [20453536](http://www.ncbi.nlm.nih.gov/pubmed/20453536) |
|  | [PA37400](http://www.pharmgkb.org/do/serve?objId=PA37400&objCls=Gene) | XBP1 | PMID: [20453536](http://www.ncbi.nlm.nih.gov/pubmed/20453536) |
|  | [PA26864](http://www.pharmgkb.org/do/serve?objId=PA26864&objCls=Gene) | CREB1 | PMID: [20453536](http://www.ncbi.nlm.nih.gov/pubmed/20453536) |
|  | [PA29861](http://www.pharmgkb.org/do/serve?objId=PA29861&objCls=Gene) | IMPA2 | PMID: [20453536](http://www.ncbi.nlm.nih.gov/pubmed/20453536) |

Table S6. The gene information for “antiinflammatory and antirheumatic products, non-steroids”

| **ID** | **PharmGKB Accession Id** | **Gene Symbol** | **Publications** |
| --- | --- | --- | --- |
|  | [PA125](http://www.pharmgkb.org/do/serve?objId=PA125&objCls=Gene) | CYP2C8 | PMID: [18216720](http://www.ncbi.nlm.nih.gov/pubmed/18216720) |
|  | [PA126](http://www.pharmgkb.org/do/serve?objId=PA126&objCls=Gene) | CYP2C9 | PMID: [18216720](http://www.ncbi.nlm.nih.gov/pubmed/18216720)[19152219](http://www.ncbi.nlm.nih.gov/pubmed/19152219) |
|  | [PA38295](http://www.pharmgkb.org/do/serve?objId=PA38295&objCls=Gene) | SLC22A11 | PMID: [12130730](http://www.ncbi.nlm.nih.gov/pubmed/12130730) |
|  | [PA388](http://www.pharmgkb.org/do/serve?objId=PA388&objCls=Gene) | SLC22A6 | PMID: [12130730](http://www.ncbi.nlm.nih.gov/pubmed/12130730) |
|  | [PA389](http://www.pharmgkb.org/do/serve?objId=PA389&objCls=Gene) | SLC22A8 | PMID: [12130730](http://www.ncbi.nlm.nih.gov/pubmed/12130730) |
|  | [PA38102](http://www.pharmgkb.org/do/serve?objId=PA38102&objCls=Gene) | SLC22A9 | PMID: [12130730](http://www.ncbi.nlm.nih.gov/pubmed/12130730) |
|  | [PA24910](http://www.pharmgkb.org/do/serve?objId=PA24910&objCls=Gene) | APP | PMID: [17082448](http://www.ncbi.nlm.nih.gov/pubmed/17082448) |
|  | [PA128394579](http://www.pharmgkb.org/do/serve?objId=PA128394579&objCls=Gene) | TMED10 | PMID: [17082448](http://www.ncbi.nlm.nih.gov/pubmed/17082448) |
|  | [PA33557](http://www.pharmgkb.org/do/serve?objId=PA33557&objCls=Gene) | PPARD | PMID: [16344721](http://www.ncbi.nlm.nih.gov/pubmed/16344721) |
|  | [PA419](http://www.pharmgkb.org/do/serve?objId=PA419&objCls=Gene) | UGT1A9 | PMID: [11927765](http://www.ncbi.nlm.nih.gov/pubmed/11927765) |
|  | [PA37178](http://www.pharmgkb.org/do/serve?objId=PA37178&objCls=Gene) | UGT1A3 | PMID: [11927765](http://www.ncbi.nlm.nih.gov/pubmed/11927765) |

Table S7. The gene information for “glatiramer acetate”

| **ID** | **PharmGKB Accession Id** | **Gene Symbol** | **Publications** |
| --- | --- | --- | --- |
|  | [PA27041](http://www.pharmgkb.org/do/serve?objId=PA27041&objCls=Gene) | CTSS | PMID: [20038765](http://www.ncbi.nlm.nih.gov/pubmed/20038765) |
|  | [PA36837](http://www.pharmgkb.org/do/serve?objId=PA36837&objCls=Gene) | TRB | PMID: [20038765](http://www.ncbi.nlm.nih.gov/pubmed/20038765) |
|  | [PA24910](http://www.pharmgkb.org/do/serve?objId=PA24910&objCls=Gene) | APP | PMID: [17082448](http://www.ncbi.nlm.nih.gov/pubmed/17082448) |
|  | [PA128394579](http://www.pharmgkb.org/do/serve?objId=PA128394579&objCls=Gene) | TMED10 | PMID: [17082448](http://www.ncbi.nlm.nih.gov/pubmed/17082448) |

Table S8. The gene information for “hmg coa reductase inhibitors”

| **ID** | **PharmGKB Accession Id** | **Gene Symbol** | **Publications** |
| --- | --- | --- | --- |
|  | [PA229](http://www.pharmgkb.org/do/serve?objId=PA229&objCls=Gene) | LEPR | PMID: [20195290](http://www.ncbi.nlm.nih.gov/pubmed/20195290) |
|  | [PA164742099](http://www.pharmgkb.org/do/serve?objId=PA164742099&objCls=Gene) | MTTP | PMID: [20195290](http://www.ncbi.nlm.nih.gov/pubmed/20195290) |
|  | [PA33529](http://www.pharmgkb.org/do/serve?objId=PA33529&objCls=Gene) | PON1 | PMID: [20195290](http://www.ncbi.nlm.nih.gov/pubmed/20195290) |
|  | [PA33557](http://www.pharmgkb.org/do/serve?objId=PA33557&objCls=Gene) | PPARD | PMID: [20195290](http://www.ncbi.nlm.nih.gov/pubmed/20195290) |
|  | [PA281](http://www.pharmgkb.org/do/serve?objId=PA281&objCls=Gene) | PPARG | PMID:[20195290](http://www.ncbi.nlm.nih.gov/pubmed/20195290) |
|  | [PA335](http://www.pharmgkb.org/do/serve?objId=PA335&objCls=Gene) | SREBF1 | PMID: [20235787](http://www.ncbi.nlm.nih.gov/pubmed/20235787) [20195290](http://www.ncbi.nlm.nih.gov/pubmed/20195290) |
|  | [PA29955](http://www.pharmgkb.org/do/serve?objId=PA29955&objCls=Gene) | ITGB2 | PMID: [21037509](http://www.ncbi.nlm.nih.gov/pubmed/21037509) |
|  | [PA36609](http://www.pharmgkb.org/do/serve?objId=PA36609&objCls=Gene) | TNFRSF1A | PMID: [21037509](http://www.ncbi.nlm.nih.gov/pubmed/21037509) |
|  | [PA24536](http://www.pharmgkb.org/do/serve?objId=PA24536&objCls=Gene) | ADAMTS1 | PMID: [21037509](http://www.ncbi.nlm.nih.gov/pubmed/21037509) |
|  | [PA131](http://www.pharmgkb.org/do/serve?objId=PA131&objCls=Gene) | CYP3A5 | PMID: [19530969](http://www.ncbi.nlm.nih.gov/pubmed/19530969) |
|  | [PA134865839](http://www.pharmgkb.org/do/serve?objId=PA134865839&objCls=Gene) | SLCO1B1 | PMID: [19884908](http://www.ncbi.nlm.nih.gov/pubmed/19884908) [18852207](http://www.ncbi.nlm.nih.gov/pubmed/18852207) [19762696](http://www.ncbi.nlm.nih.gov/pubmed/19762696) [20712525](http://www.ncbi.nlm.nih.gov/pubmed/20712525) [19833261](http://www.ncbi.nlm.nih.gov/pubmed/19833261) |
|  | [PA267](http://www.pharmgkb.org/do/serve?objId=PA267&objCls=Gene) | ABCB1 | PMID: [18622260](http://www.ncbi.nlm.nih.gov/pubmed/18622260) [20712525](http://www.ncbi.nlm.nih.gov/pubmed/20712525) |
|  | [PA108](http://www.pharmgkb.org/do/serve?objId=PA108&objCls=Gene) | CETP | PMID:[20195290](http://www.ncbi.nlm.nih.gov/pubmed/20195290) [18622260](http://www.ncbi.nlm.nih.gov/pubmed/18622260) [20195290](http://www.ncbi.nlm.nih.gov/pubmed/20195290) |
|  | [PA227](http://www.pharmgkb.org/do/serve?objId=PA227&objCls=Gene) | LDLR | PMID: [18622260](http://www.ncbi.nlm.nih.gov/pubmed/18622260) [20195290](http://www.ncbi.nlm.nih.gov/pubmed/20195290) |
|  | [PA230](http://www.pharmgkb.org/do/serve?objId=PA230&objCls=Gene) | LIPC | PMID: [18622260](http://www.ncbi.nlm.nih.gov/pubmed/18622260) [20195290](http://www.ncbi.nlm.nih.gov/pubmed/20195290) |
|  | [PA254](http://www.pharmgkb.org/do/serve?objId=PA254&objCls=Gene) | NOS3 | PMID: [18622260](http://www.ncbi.nlm.nih.gov/pubmed/18622260) |
|  | [PA24422](http://www.pharmgkb.org/do/serve?objId=PA24422&objCls=Gene) | ACACB | PMID: [20602615](http://www.ncbi.nlm.nih.gov/pubmed/20602615) |
|  | [PA50](http://www.pharmgkb.org/do/serve?objId=PA50&objCls=Gene) | APOB | PMID: [20195290](http://www.ncbi.nlm.nih.gov/pubmed/20195290) |
|  | [PA51](http://www.pharmgkb.org/do/serve?objId=PA51&objCls=Gene) | APOC1 | PMID: [20339536](http://www.ncbi.nlm.nih.gov/pubmed/20339536) |
|  | [PA132](http://www.pharmgkb.org/do/serve?objId=PA132&objCls=Gene) | CYP7A1 | PMID: [20195290](http://www.ncbi.nlm.nih.gov/pubmed/20195290) |
|  | [PA28073](http://www.pharmgkb.org/do/serve?objId=PA28073&objCls=Gene) | FDFT1 | PMID: [20195290](http://www.ncbi.nlm.nih.gov/pubmed/20195290) |
|  | [PA232](http://www.pharmgkb.org/do/serve?objId=PA232&objCls=Gene) | LPL | PMID: [20195290](http://www.ncbi.nlm.nih.gov/pubmed/20195290) |
|  | [PA24373](http://www.pharmgkb.org/do/serve?objId=PA24373&objCls=Gene) | ABCA1 | PMID: [20195290](http://www.ncbi.nlm.nih.gov/pubmed/20195290) |
|  | [PA24412](http://www.pharmgkb.org/do/serve?objId=PA24412&objCls=Gene) | ABCG8 | PMID: [20235787](http://www.ncbi.nlm.nih.gov/pubmed/20235787) [20195290](http://www.ncbi.nlm.nih.gov/pubmed/20195290) |
|  | [PA49](http://www.pharmgkb.org/do/serve?objId=PA49&objCls=Gene) | APOA1 | PMID: [20195290](http://www.ncbi.nlm.nih.gov/pubmed/20195290) [20195290](http://www.ncbi.nlm.nih.gov/pubmed/20195290) |
|  | [PA37253](http://www.pharmgkb.org/do/serve?objId=PA37253&objCls=Gene) | USP5 | PMID: none |
|  | [PA139](http://www.pharmgkb.org/do/serve?objId=PA139&objCls=Gene) | ACE | PMID: [17184205](http://www.ncbi.nlm.nih.gov/pubmed/17184205) |
|  | [PA55](http://www.pharmgkb.org/do/serve?objId=PA55&objCls=Gene) | APOE | PMID: [18388879](http://www.ncbi.nlm.nih.gov/pubmed/18388879) [19529002](http://www.ncbi.nlm.nih.gov/pubmed/19529002) [20195290](http://www.ncbi.nlm.nih.gov/pubmed/20195290) [15530894](http://www.ncbi.nlm.nih.gov/pubmed/15530894) [18334912](http://www.ncbi.nlm.nih.gov/pubmed/18334912) |
|  | [PA35752](http://www.pharmgkb.org/do/serve?objId=PA35752&objCls=Gene) | SHH | PMID: [15546153](http://www.ncbi.nlm.nih.gov/pubmed/15546153) |
|  | [PA189](http://www.pharmgkb.org/do/serve?objId=PA189&objCls=Gene) | HMGCR | PMID: [20005478](http://www.ncbi.nlm.nih.gov/pubmed/20005478) [20403997](http://www.ncbi.nlm.nih.gov/pubmed/20403997) [18332269](http://www.ncbi.nlm.nih.gov/pubmed/18332269) [15488882](http://www.ncbi.nlm.nih.gov/pubmed/15488882) [18622260](http://www.ncbi.nlm.nih.gov/pubmed/18622260) [20235787](http://www.ncbi.nlm.nih.gov/pubmed/20235787) [20195290](http://www.ncbi.nlm.nih.gov/pubmed/20195290) [18815589](http://www.ncbi.nlm.nih.gov/pubmed/18815589) |
|  | [PA293](http://www.pharmgkb.org/do/serve?objId=PA293&objCls=Gene) | PTGS2 | PMID: [15530865](http://www.ncbi.nlm.nih.gov/pubmed/15530865) |
|  | [PA130](http://www.pharmgkb.org/do/serve?objId=PA130&objCls=Gene) | CYP3A4 | PMID: [20386561](http://www.ncbi.nlm.nih.gov/pubmed/20386561) [15518608](http://www.ncbi.nlm.nih.gov/pubmed/15518608) [19530969](http://www.ncbi.nlm.nih.gov/pubmed/19530969) [16563062](http://www.ncbi.nlm.nih.gov/pubmed/16563062) [19833261](http://www.ncbi.nlm.nih.gov/pubmed/19833261) |
|  | [PA120](http://www.pharmgkb.org/do/serve?objId=PA120&objCls=Gene) | CRP | PMID: [15635109](http://www.ncbi.nlm.nih.gov/pubmed/15635109) [12595863](http://www.ncbi.nlm.nih.gov/pubmed/12595863) [11952885](http://www.ncbi.nlm.nih.gov/pubmed/11952885) |
|  | [PA36552](http://www.pharmgkb.org/do/serve?objId=PA36552&objCls=Gene) | TLR4 | PMID: [15864121](http://www.ncbi.nlm.nih.gov/pubmed/15864121) |
|  | [PA134865095](http://www.pharmgkb.org/do/serve?objId=PA134865095&objCls=Gene) | RHOA | PMID: [15932062](http://www.ncbi.nlm.nih.gov/pubmed/15932062) |
|  | [PA176](http://www.pharmgkb.org/do/serve?objId=PA176&objCls=Gene) | GNB3 | PMID: [18551043](http://www.ncbi.nlm.nih.gov/pubmed/18551043) |
|  | [PA38617](http://www.pharmgkb.org/do/serve?objId=PA38617&objCls=Gene) | PCSK9 | PMID: [16424354](http://www.ncbi.nlm.nih.gov/pubmed/16424354) |
|  | [PA134945356](http://www.pharmgkb.org/do/serve?objId=PA134945356&objCls=Gene) | CLMN | PMID: [20339536](http://www.ncbi.nlm.nih.gov/pubmed/20339536) |
|  | [PA147031775](http://www.pharmgkb.org/do/serve?objId=PA147031775&objCls=Gene) | KIAA0999 | PMID: none |

Table S9. The gene information for “curcumin”

| **ID** | **PharmGKB Accession Id** | **Gene Symbol** | **Publications** |
| --- | --- | --- | --- |
|  | [PA26866](http://www.pharmgkb.org/do/serve?objId=PA26866&objCls=Gene) | CREBBP | PMID: [19577000](http://www.ncbi.nlm.nih.gov/pubmed/19577000) |
|  | [PA27807](http://www.pharmgkb.org/do/serve?objId=PA27807&objCls=Gene) | EP300 | PMID: [19577000](http://www.ncbi.nlm.nih.gov/pubmed/19577000) |

Table S10. The gene information for “vitamin c”

| **ID** | **PharmGKB Accession Id** | **Gene Symbol** | **Publications** |
| --- | --- | --- | --- |
|  | [PA27068](http://www.pharmgkb.org/do/serve?objId=PA27068&objCls=Gene) | CYB5A | PMID: none |
|  | [PA27331](http://www.pharmgkb.org/do/serve?objId=PA27331&objCls=Gene) | CYB5R3 | PMID: none |
|  | [PA26826](http://www.pharmgkb.org/do/serve?objId=PA26826&objCls=Gene) | CPLX1 | PMID: none |
|  | [PA236](http://www.pharmgkb.org/do/serve?objId=PA236&objCls=Gene) | MAOA | PMID: none |
|  | [PA34132](http://www.pharmgkb.org/do/serve?objId=PA34132&objCls=Gene) | RAB3A | PMID: none |
|  | [PA38220](http://www.pharmgkb.org/do/serve?objId=PA38220&objCls=Gene) | RIMS1 | PMID: none |
|  | [PA325](http://www.pharmgkb.org/do/serve?objId=PA325&objCls=Gene) | SLC18A2 | PMID: none |
|  | [PA331](http://www.pharmgkb.org/do/serve?objId=PA331&objCls=Gene) | SLC22A2 | PMID: none |
|  | [PA35980](http://www.pharmgkb.org/do/serve?objId=PA35980&objCls=Gene) | SNAP25 | PMID: none |
|  | [PA36233](http://www.pharmgkb.org/do/serve?objId=PA36233&objCls=Gene) | STX1A | PMID: none |
|  | [PA36241](http://www.pharmgkb.org/do/serve?objId=PA36241&objCls=Gene) | STXBP1 | PMID: none |
|  | [PA36290](http://www.pharmgkb.org/do/serve?objId=PA36290&objCls=Gene) | SYT1 | PMID: none |
|  | [PA37267](http://www.pharmgkb.org/do/serve?objId=PA37267&objCls=Gene) | VAMP2 | PMID: none |
|  | [PA140](http://www.pharmgkb.org/do/serve?objId=PA140&objCls=Gene) | DDC | PMID: none |
|  | [PA274](http://www.pharmgkb.org/do/serve?objId=PA274&objCls=Gene) | PNMT | PMID: none |
|  | [PA136](http://www.pharmgkb.org/do/serve?objId=PA136&objCls=Gene) | DBH | PMID: none |

Table S11. The gene information for “vitamin e”

| **ID** | **PharmGKB Accession Id** | **Gene Symbol** | **Publications** |
| --- | --- | --- | --- |
|  | [**PA29415**](http://www.pharmgkb.org/do/serve?objId=PA29415&objCls=Gene) | HP | PMID: [20415560](http://www.ncbi.nlm.nih.gov/pubmed/20415560) |

Table S12. The gene information for “antidepressants”

| **ID** | **PharmGKB Accession Id** | **Gene Symbol** | **Publications** |
| --- | --- | --- | --- |
|  | [PA27660](http://www.pharmgkb.org/do/serve?objId=PA27660&objCls=Gene) | EFNA5 | PMID: [19736353](http://www.ncbi.nlm.nih.gov/pubmed/19736353) |
|  | [PA27821](http://www.pharmgkb.org/do/serve?objId=PA27821&objCls=Gene) | EPHA5 | PMID: [19736353](http://www.ncbi.nlm.nih.gov/pubmed/19736353) |
|  | [PA31756](http://www.pharmgkb.org/do/serve?objId=PA31756&objCls=Gene) | NR2E1 | PMID: [19736353](http://www.ncbi.nlm.nih.gov/pubmed/19736353) |
|  | [PA134972608](http://www.pharmgkb.org/do/serve?objId=PA134972608&objCls=Gene) | HOMER1 | PMID: [19736353](http://www.ncbi.nlm.nih.gov/pubmed/19736353) |
|  | [PA31776](http://www.pharmgkb.org/do/serve?objId=PA31776&objCls=Gene) | NRG1 | PMID: [19736353](http://www.ncbi.nlm.nih.gov/pubmed/19736353) |
|  | [PA35827](http://www.pharmgkb.org/do/serve?objId=PA35827&objCls=Gene) | SLC1A2 | PMID: [19736353](http://www.ncbi.nlm.nih.gov/pubmed/19736353) |
|  | [PA35907](http://www.pharmgkb.org/do/serve?objId=PA35907&objCls=Gene) | SLC6A11 | PMID: [19736353](http://www.ncbi.nlm.nih.gov/pubmed/19736353) |
|  | [PA35](http://www.pharmgkb.org/do/serve?objId=PA35&objCls=Gene) | ADRA2A | PMID: [18568127](http://www.ncbi.nlm.nih.gov/pubmed/18568127) |
|  | [PA38](http://www.pharmgkb.org/do/serve?objId=PA38&objCls=Gene) | ADRB1 | PMID: [18568127](http://www.ncbi.nlm.nih.gov/pubmed/18568127) |
|  | [PA55](http://www.pharmgkb.org/do/serve?objId=PA55&objCls=Gene) | APOE | PMID: [18568127](http://www.ncbi.nlm.nih.gov/pubmed/18568127) |
|  | [PA117](http://www.pharmgkb.org/do/serve?objId=PA117&objCls=Gene) | COMT | PMID: [20047055](http://www.ncbi.nlm.nih.gov/pubmed/20047055) [18568127](http://www.ncbi.nlm.nih.gov/pubmed/18568127) |
|  | [PA26875](http://www.pharmgkb.org/do/serve?objId=PA26875&objCls=Gene) | CRHR2 | PMID: [18568127](http://www.ncbi.nlm.nih.gov/pubmed/18568127) |
|  | [PA27478](http://www.pharmgkb.org/do/serve?objId=PA27478&objCls=Gene) | DRD2 | PMID: [16633151](http://www.ncbi.nlm.nih.gov/pubmed/16633151) [18568127](http://www.ncbi.nlm.nih.gov/pubmed/18568127) |
|  | [PA175](http://www.pharmgkb.org/do/serve?objId=PA175&objCls=Gene) | GNAS | PMID: [18568127](http://www.ncbi.nlm.nih.gov/pubmed/18568127) |
|  | [PA29549](http://www.pharmgkb.org/do/serve?objId=PA29549&objCls=Gene) | HTR1B | PMID: [18568127](http://www.ncbi.nlm.nih.gov/pubmed/18568127) |
|  | [PA194](http://www.pharmgkb.org/do/serve?objId=PA194&objCls=Gene) | HTR2C | PMID: [18568127](http://www.ncbi.nlm.nih.gov/pubmed/18568127) |
|  | [PA29555](http://www.pharmgkb.org/do/serve?objId=PA29555&objCls=Gene) | HTR3A | PMID: [19558256](http://www.ncbi.nlm.nih.gov/pubmed/19558256) [18568127](http://www.ncbi.nlm.nih.gov/pubmed/18568127) |
|  | [PA29560](http://www.pharmgkb.org/do/serve?objId=PA29560&objCls=Gene) | HTR6 | PMID: [18568127](http://www.ncbi.nlm.nih.gov/pubmed/18568127) |
|  | [PA236](http://www.pharmgkb.org/do/serve?objId=PA236&objCls=Gene) | MAOA | PMID: [19558256](http://www.ncbi.nlm.nih.gov/pubmed/19558256) [18568127](http://www.ncbi.nlm.nih.gov/pubmed/18568127) |
|  | [PA245](http://www.pharmgkb.org/do/serve?objId=PA245&objCls=Gene) | MTHFR | PMID: [18568127](http://www.ncbi.nlm.nih.gov/pubmed/18568127) |
|  | [PA252](http://www.pharmgkb.org/do/serve?objId=PA252&objCls=Gene) | NOS1 | PMID: [18568127](http://www.ncbi.nlm.nih.gov/pubmed/18568127) |
|  | [PA310](http://www.pharmgkb.org/do/serve?objId=PA310&objCls=Gene) | SLC6A2 | PMID: [18568127](http://www.ncbi.nlm.nih.gov/pubmed/18568127) [17714023](http://www.ncbi.nlm.nih.gov/pubmed/17714023) [20047055](http://www.ncbi.nlm.nih.gov/pubmed/20047055) |
|  | [PA26864](http://www.pharmgkb.org/do/serve?objId=PA26864&objCls=Gene) | CREB1 | PMID: [19558256](http://www.ncbi.nlm.nih.gov/pubmed/19558256) |
|  | [PA29556](http://www.pharmgkb.org/do/serve?objId=PA29556&objCls=Gene) | HTR3B | PMID: [19558256](http://www.ncbi.nlm.nih.gov/pubmed/19558256) |
|  | [PA33128](http://www.pharmgkb.org/do/serve?objId=PA33128&objCls=Gene) | PDE4A | PMID: [19442182](http://www.ncbi.nlm.nih.gov/pubmed/19442182) |
|  | [PA25335](http://www.pharmgkb.org/do/serve?objId=PA25335&objCls=Gene) | BDNF | PMID: [20195291](http://www.ncbi.nlm.nih.gov/pubmed/20195291) [19414708](http://www.ncbi.nlm.nih.gov/pubmed/19414708) [20047055](http://www.ncbi.nlm.nih.gov/pubmed/20047055) [18568127](http://www.ncbi.nlm.nih.gov/pubmed/18568127) [19403460](http://www.ncbi.nlm.nih.gov/pubmed/19403460) |
|  | [PA26874](http://www.pharmgkb.org/do/serve?objId=PA26874&objCls=Gene) | CRHR1 | PMID: [19403460](http://www.ncbi.nlm.nih.gov/pubmed/19403460) |
|  | [PA28162](http://www.pharmgkb.org/do/serve?objId=PA28162&objCls=Gene) | FKBP5 | PMID: [16581694](http://www.ncbi.nlm.nih.gov/pubmed/16581694) [20102306](http://www.ncbi.nlm.nih.gov/pubmed/20102306) [20047055](http://www.ncbi.nlm.nih.gov/pubmed/20047055) [19403460](http://www.ncbi.nlm.nih.gov/pubmed/19403460) |
|  | [PA192](http://www.pharmgkb.org/do/serve?objId=PA192&objCls=Gene) | HTR1A | PMID: [20047055](http://www.ncbi.nlm.nih.gov/pubmed/20047055) [18568127](http://www.ncbi.nlm.nih.gov/pubmed/18568127) [15212588](http://www.ncbi.nlm.nih.gov/pubmed/15212588) [19403460](http://www.ncbi.nlm.nih.gov/pubmed/19403460) |
|  | [PA193](http://www.pharmgkb.org/do/serve?objId=PA193&objCls=Gene) | HTR2A | PMID: [16581694](http://www.ncbi.nlm.nih.gov/pubmed/16581694) [19558256](http://www.ncbi.nlm.nih.gov/pubmed/19558256) [20102306](http://www.ncbi.nlm.nih.gov/pubmed/20102306) [20047055](http://www.ncbi.nlm.nih.gov/pubmed/20047055) [18568127](http://www.ncbi.nlm.nih.gov/pubmed/18568127) [19403460](http://www.ncbi.nlm.nih.gov/pubmed/19403460) |
|  | [PA267](http://www.pharmgkb.org/do/serve?objId=PA267&objCls=Gene) | ABCB1 | PMID: [19018726](http://www.ncbi.nlm.nih.gov/pubmed/19018726) |
|  | [PA28968](http://www.pharmgkb.org/do/serve?objId=PA28968&objCls=Gene) | GRIA3 | PMID: [19018726](http://www.ncbi.nlm.nih.gov/pubmed/19018726) |
|  | [PA28976](http://www.pharmgkb.org/do/serve?objId=PA28976&objCls=Gene) | GRIK4 | PMID: [19018726](http://www.ncbi.nlm.nih.gov/pubmed/19018726) |
|  | [PA28977](http://www.pharmgkb.org/do/serve?objId=PA28977&objCls=Gene) | GRIK5 | PMID: [19018726](http://www.ncbi.nlm.nih.gov/pubmed/19018726) |
|  | [PA176](http://www.pharmgkb.org/do/serve?objId=PA176&objCls=Gene) | GNB3 | PMID: [16581694](http://www.ncbi.nlm.nih.gov/pubmed/16581694) [18568127](http://www.ncbi.nlm.nih.gov/pubmed/18568127) [10884039](http://www.ncbi.nlm.nih.gov/pubmed/10884039) |
|  | [PA311](http://www.pharmgkb.org/do/serve?objId=PA311&objCls=Gene) | SLC6A3 | PMID: [16702979](http://www.ncbi.nlm.nih.gov/pubmed/16702979) [18568127](http://www.ncbi.nlm.nih.gov/pubmed/18568127) [17714023](http://www.ncbi.nlm.nih.gov/pubmed/17714023) |
|  | [PA312](http://www.pharmgkb.org/do/serve?objId=PA312&objCls=Gene) | SLC6A4 | PMID: [12746735](http://www.ncbi.nlm.nih.gov/pubmed/12746735) [19558256](http://www.ncbi.nlm.nih.gov/pubmed/19558256) [16702979](http://www.ncbi.nlm.nih.gov/pubmed/16702979) [15037864](http://www.ncbi.nlm.nih.gov/pubmed/15037864) [19018726](http://www.ncbi.nlm.nih.gov/pubmed/19018726) [16515395](http://www.ncbi.nlm.nih.gov/pubmed/16515395) [16581694](http://www.ncbi.nlm.nih.gov/pubmed/16581694) [19568851](http://www.ncbi.nlm.nih.gov/pubmed/19568851) [18855611](http://www.ncbi.nlm.nih.gov/pubmed/18855611) [20102306](http://www.ncbi.nlm.nih.gov/pubmed/20102306) [18568127](http://www.ncbi.nlm.nih.gov/pubmed/18568127) [17714023](http://www.ncbi.nlm.nih.gov/pubmed/17714023) [20047055](http://www.ncbi.nlm.nih.gov/pubmed/20047055) [19403460](http://www.ncbi.nlm.nih.gov/pubmed/19403460) |
|  | [PA119](http://www.pharmgkb.org/do/serve?objId=PA119&objCls=Gene) | CRH | PMID: [14735130](http://www.ncbi.nlm.nih.gov/pubmed/14735130) [18568127](http://www.ncbi.nlm.nih.gov/pubmed/18568127) |
|  | [PA355](http://www.pharmgkb.org/do/serve?objId=PA355&objCls=Gene) | TPH1 | PMID: [16581694](http://www.ncbi.nlm.nih.gov/pubmed/16581694) [20047055](http://www.ncbi.nlm.nih.gov/pubmed/20047055) [18568127](http://www.ncbi.nlm.nih.gov/pubmed/18568127) [15111987](http://www.ncbi.nlm.nih.gov/pubmed/15111987) |
|  | [PA128](http://www.pharmgkb.org/do/serve?objId=PA128&objCls=Gene) | CYP2D6 | PMID: [16581694](http://www.ncbi.nlm.nih.gov/pubmed/16581694) [19059066](http://www.ncbi.nlm.nih.gov/pubmed/19059066) [18691982](http://www.ncbi.nlm.nih.gov/pubmed/18691982) [19924131](http://www.ncbi.nlm.nih.gov/pubmed/19924131) [16706732](http://www.ncbi.nlm.nih.gov/pubmed/16706732) [15116051](http://www.ncbi.nlm.nih.gov/pubmed/15116051) [7903915](http://www.ncbi.nlm.nih.gov/pubmed/7903915) [19320528](http://www.ncbi.nlm.nih.gov/pubmed/19320528) [15168101](http://www.ncbi.nlm.nih.gov/pubmed/15168101) [10688272](http://www.ncbi.nlm.nih.gov/pubmed/10688272) [18800072](http://www.ncbi.nlm.nih.gov/pubmed/18800072) |
|  | [PA124](http://www.pharmgkb.org/do/serve?objId=PA124&objCls=Gene) | CYP2C19 | PMID: [19059066](http://www.ncbi.nlm.nih.gov/pubmed/19059066) [16706732](http://www.ncbi.nlm.nih.gov/pubmed/16706732) [15168101](http://www.ncbi.nlm.nih.gov/pubmed/15168101) [15037866](http://www.ncbi.nlm.nih.gov/pubmed/15037866) [18800072](http://www.ncbi.nlm.nih.gov/pubmed/18800072) |
|  | [PA26609](http://www.pharmgkb.org/do/serve?objId=PA26609&objCls=Gene) | CLOCK | PMID: [15475734](http://www.ncbi.nlm.nih.gov/pubmed/15475734) |
|  | [PA128747823](http://www.pharmgkb.org/do/serve?objId=PA128747823&objCls=Gene) | TPH2 | PMID: [20047055](http://www.ncbi.nlm.nih.gov/pubmed/20047055) [18496129](http://www.ncbi.nlm.nih.gov/pubmed/18496129) |

Table S13. The gene information for “antipsychotics”

| **ID** | **PharmGKB Accession Id** | **Gene Symbol** | **Publications** |
| --- | --- | --- | --- |
|  | [PA26681](http://www.pharmgkb.org/do/serve?objId=PA26681&objCls=Gene) | CNR1 | PMID: [21266946](http://www.ncbi.nlm.nih.gov/pubmed/21266946) |
|  | [PA147](http://www.pharmgkb.org/do/serve?objId=PA147&objCls=Gene) | DRD1 | PMID: [20714340](http://www.ncbi.nlm.nih.gov/pubmed/20714340) |
|  | [PA148](http://www.pharmgkb.org/do/serve?objId=PA148&objCls=Gene) | DRD5 | PMID: [20714340](http://www.ncbi.nlm.nih.gov/pubmed/20714340) |
|  | [PA134872551](http://www.pharmgkb.org/do/serve?objId=PA134872551&objCls=Gene) | ANKK1 | PMID: none |
|  | [PA27512](http://www.pharmgkb.org/do/serve?objId=PA27512&objCls=Gene) | DTNBP1 | PMID: [18698228](http://www.ncbi.nlm.nih.gov/pubmed/18698228) |
|  | [PA27779](http://www.pharmgkb.org/do/serve?objId=PA27779&objCls=Gene) | EN1 | PMID: [18698228](http://www.ncbi.nlm.nih.gov/pubmed/18698228) |
|  | [PA35018](http://www.pharmgkb.org/do/serve?objId=PA35018&objCls=Gene) | SCTR | PMID: [18698228](http://www.ncbi.nlm.nih.gov/pubmed/18698228) |
|  | [PA29556](http://www.pharmgkb.org/do/serve?objId=PA29556&objCls=Gene) | HTR3B | PMID: [18807291](http://www.ncbi.nlm.nih.gov/pubmed/18807291) |
|  | [PA30584](http://www.pharmgkb.org/do/serve?objId=PA30584&objCls=Gene) | MAP2K1 | PMID: [18319075](http://www.ncbi.nlm.nih.gov/pubmed/18319075) |
|  | [PA83](http://www.pharmgkb.org/do/serve?objId=PA83&objCls=Gene) | CACNA1C | PMID: [21079043](http://www.ncbi.nlm.nih.gov/pubmed/21079043) |
|  | [PA223](http://www.pharmgkb.org/do/serve?objId=PA223&objCls=Gene) | KCNQ1 | PMID: [21079043](http://www.ncbi.nlm.nih.gov/pubmed/21079043) |
|  | [PA35007](http://www.pharmgkb.org/do/serve?objId=PA35007&objCls=Gene) | SCN4B | PMID: [21079043](http://www.ncbi.nlm.nih.gov/pubmed/21079043) |
|  | [PA304](http://www.pharmgkb.org/do/serve?objId=PA304&objCls=Gene) | SCN5A | PMID: [21079043](http://www.ncbi.nlm.nih.gov/pubmed/21079043) |
|  | [PA36007](http://www.pharmgkb.org/do/serve?objId=PA36007&objCls=Gene) | SNTA1 | PMID: [21079043](http://www.ncbi.nlm.nih.gov/pubmed/21079043) |
|  | [PA26109](http://www.pharmgkb.org/do/serve?objId=PA26109&objCls=Gene) | CAV3 | PMID: [21079043](http://www.ncbi.nlm.nih.gov/pubmed/21079043) |
|  | [PA211](http://www.pharmgkb.org/do/serve?objId=PA211&objCls=Gene) | KCNE1 | PMID: [21079043](http://www.ncbi.nlm.nih.gov/pubmed/21079043) |
|  | [PA392](http://www.pharmgkb.org/do/serve?objId=PA392&objCls=Gene) | KCNE2 | PMID: [21079043](http://www.ncbi.nlm.nih.gov/pubmed/21079043) |
|  | [PA212](http://www.pharmgkb.org/do/serve?objId=PA212&objCls=Gene) | KCNH2 | PMID: [21079043](http://www.ncbi.nlm.nih.gov/pubmed/21079043) |
|  | [PA214](http://www.pharmgkb.org/do/serve?objId=PA214&objCls=Gene) | KCNJ2 | PMID: [21079043](http://www.ncbi.nlm.nih.gov/pubmed/21079043) |
|  | [PA216](http://www.pharmgkb.org/do/serve?objId=PA216&objCls=Gene) | KCNJ5 | PMID: [21079043](http://www.ncbi.nlm.nih.gov/pubmed/21079043) |
|  | [PA24673](http://www.pharmgkb.org/do/serve?objId=PA24673&objCls=Gene) | AKAP9 | PMID: [21079043](http://www.ncbi.nlm.nih.gov/pubmed/21079043) |
|  | [PA24799](http://www.pharmgkb.org/do/serve?objId=PA24799&objCls=Gene) | ANK2 | PMID: [21079043](http://www.ncbi.nlm.nih.gov/pubmed/21079043) |
|  | [PA29555](http://www.pharmgkb.org/do/serve?objId=PA29555&objCls=Gene) | HTR3A | PMID: [19794330](http://www.ncbi.nlm.nih.gov/pubmed/19794330) |
|  | [PA134900226](http://www.pharmgkb.org/do/serve?objId=PA134900226&objCls=Gene) | HTR3E | PMID: [19794330](http://www.ncbi.nlm.nih.gov/pubmed/19794330) |
|  | [PA117](http://www.pharmgkb.org/do/serve?objId=PA117&objCls=Gene) | COMT | PMID: [15115916](http://www.ncbi.nlm.nih.gov/pubmed/15115916) [19506579](http://www.ncbi.nlm.nih.gov/pubmed/19506579) [15465976](http://www.ncbi.nlm.nih.gov/pubmed/15465976) [12729939](http://www.ncbi.nlm.nih.gov/pubmed/12729939) [17363961](http://www.ncbi.nlm.nih.gov/pubmed/17363961) [16702905](http://www.ncbi.nlm.nih.gov/pubmed/16702905) [15522252](http://www.ncbi.nlm.nih.gov/pubmed/15522252) [14520117](http://www.ncbi.nlm.nih.gov/pubmed/14520117) [12815736](http://www.ncbi.nlm.nih.gov/pubmed/12815736) |
|  | [PA236](http://www.pharmgkb.org/do/serve?objId=PA236&objCls=Gene) | MAOA | PMID: [19506579](http://www.ncbi.nlm.nih.gov/pubmed/19506579) [16702905](http://www.ncbi.nlm.nih.gov/pubmed/16702905) [14520117](http://www.ncbi.nlm.nih.gov/pubmed/14520117) |
|  | [PA139](http://www.pharmgkb.org/do/serve?objId=PA139&objCls=Gene) | ACE | PMID: [19506579](http://www.ncbi.nlm.nih.gov/pubmed/19506579) [12729939](http://www.ncbi.nlm.nih.gov/pubmed/12729939) |
|  | [PA31686](http://www.pharmgkb.org/do/serve?objId=PA31686&objCls=Gene) | NOTCH4 | PMID: [15115916](http://www.ncbi.nlm.nih.gov/pubmed/15115916) |
|  | [PA193](http://www.pharmgkb.org/do/serve?objId=PA193&objCls=Gene) | HTR2A | PMID: [10212557](http://www.ncbi.nlm.nih.gov/pubmed/10212557) [16314884](http://www.ncbi.nlm.nih.gov/pubmed/16314884) |
|  | [PA25335](http://www.pharmgkb.org/do/serve?objId=PA25335&objCls=Gene) | BDNF | PMID: [18408624](http://www.ncbi.nlm.nih.gov/pubmed/18408624) |
|  | [PA128](http://www.pharmgkb.org/do/serve?objId=PA128&objCls=Gene) | CYP2D6 | PMID: [15861039](http://www.ncbi.nlm.nih.gov/pubmed/15861039) [16706732](http://www.ncbi.nlm.nih.gov/pubmed/16706732) [18551040](http://www.ncbi.nlm.nih.gov/pubmed/18551040) [12432967](http://www.ncbi.nlm.nih.gov/pubmed/12432967) |
|  | [PA130](http://www.pharmgkb.org/do/serve?objId=PA130&objCls=Gene) | CYP3A4 | PMID: [18551040](http://www.ncbi.nlm.nih.gov/pubmed/18551040) |
|  | [PA131](http://www.pharmgkb.org/do/serve?objId=PA131&objCls=Gene) | CYP3A5 | PMID: [18551040](http://www.ncbi.nlm.nih.gov/pubmed/18551040) |
|  | [PA27478](http://www.pharmgkb.org/do/serve?objId=PA27478&objCls=Gene) | DRD2 | PMID: [20714340](http://www.ncbi.nlm.nih.gov/pubmed/20714340) [20736885](http://www.ncbi.nlm.nih.gov/pubmed/20736885) [19506579](http://www.ncbi.nlm.nih.gov/pubmed/19506579) [20664489](http://www.ncbi.nlm.nih.gov/pubmed/20664489) [18551040](http://www.ncbi.nlm.nih.gov/pubmed/18551040) [20375926](http://www.ncbi.nlm.nih.gov/pubmed/20375926) [20194480](http://www.ncbi.nlm.nih.gov/pubmed/20194480) [20714340](http://www.ncbi.nlm.nih.gov/pubmed/20714340) [20102306](http://www.ncbi.nlm.nih.gov/pubmed/20102306) [19763115](http://www.ncbi.nlm.nih.gov/pubmed/19763115) |
|  | [PA254](http://www.pharmgkb.org/do/serve?objId=PA254&objCls=Gene) | NOS3 | PMID: [16495774](http://www.ncbi.nlm.nih.gov/pubmed/16495774) |
|  | [PA162397442](http://www.pharmgkb.org/do/serve?objId=PA162397442&objCls=Gene) | NEFM | PMID: [16734940](http://www.ncbi.nlm.nih.gov/pubmed/16734940) |
|  | [PA124](http://www.pharmgkb.org/do/serve?objId=PA124&objCls=Gene) | CYP2C19 | PMID: [16706732](http://www.ncbi.nlm.nih.gov/pubmed/16706732) |
|  | [PA27093](http://www.pharmgkb.org/do/serve?objId=PA27093&objCls=Gene) | CYP1A2 | PMID: [16969362](http://www.ncbi.nlm.nih.gov/pubmed/16969362) |
|  | [PA28867](http://www.pharmgkb.org/do/serve?objId=PA28867&objCls=Gene) | MCHR1 | PMID: [16983399](http://www.ncbi.nlm.nih.gov/pubmed/16983399) |
|  | [PA194](http://www.pharmgkb.org/do/serve?objId=PA194&objCls=Gene) | HTR2C | PMID: [20680028](http://www.ncbi.nlm.nih.gov/pubmed/20680028) [17016522](http://www.ncbi.nlm.nih.gov/pubmed/17016522) |
|  | [PA27479](http://www.pharmgkb.org/do/serve?objId=PA27479&objCls=Gene) | DRD3 | PMID: [20714340](http://www.ncbi.nlm.nih.gov/pubmed/20714340) [19506579](http://www.ncbi.nlm.nih.gov/pubmed/19506579) [18551040](http://www.ncbi.nlm.nih.gov/pubmed/18551040) [20102306](http://www.ncbi.nlm.nih.gov/pubmed/20102306) [9106238](http://www.ncbi.nlm.nih.gov/pubmed/9106238) [10379516](http://www.ncbi.nlm.nih.gov/pubmed/10379516) |
|  | [PA27480](http://www.pharmgkb.org/do/serve?objId=PA27480&objCls=Gene) | DRD4 | PMID: [10889553](http://www.ncbi.nlm.nih.gov/pubmed/10889553) [18332898](http://www.ncbi.nlm.nih.gov/pubmed/18332898) [20714340](http://www.ncbi.nlm.nih.gov/pubmed/20714340) |
|  | [PA34](http://www.pharmgkb.org/do/serve?objId=PA34&objCls=Gene) | ADRA1A | PMID: [19918262](http://www.ncbi.nlm.nih.gov/pubmed/19918262) |
|  | [PA245](http://www.pharmgkb.org/do/serve?objId=PA245&objCls=Gene) | MTHFR | PMID: [17976958](http://www.ncbi.nlm.nih.gov/pubmed/17976958) |
|  | [PA237](http://www.pharmgkb.org/do/serve?objId=PA237&objCls=Gene) | MAOB | PMID: [19506579](http://www.ncbi.nlm.nih.gov/pubmed/19506579) |
|  | [PA311](http://www.pharmgkb.org/do/serve?objId=PA311&objCls=Gene) | SLC6A3 | PMID: [19506579](http://www.ncbi.nlm.nih.gov/pubmed/19506579) |

Table S14. The gene information for “galantamine”

| **ID** | **PharmGKB Accession Id** | **Gene Symbol** | **Publications** |
| --- | --- | --- | --- |
|  | [PA20](http://www.pharmgkb.org/do/serve?objId=PA20&objCls=Gene) | ACHE | PMID: [14674789](http://www.ncbi.nlm.nih.gov/pubmed/14674789) [12177686](http://www.ncbi.nlm.nih.gov/pubmed/12177686) |
|  | [PA26490](http://www.pharmgkb.org/do/serve?objId=PA26490&objCls=Gene) | CHRNA4 | PMID: [14674789](http://www.ncbi.nlm.nih.gov/pubmed/14674789) [12177686](http://www.ncbi.nlm.nih.gov/pubmed/12177686) |
|  | [PA25294](http://www.pharmgkb.org/do/serve?objId=PA25294&objCls=Gene) | BCHE | PMID: [20644562](http://www.ncbi.nlm.nih.gov/pubmed/20644562) [12177686](http://www.ncbi.nlm.nih.gov/pubmed/12177686) |
|  | [PA115](http://www.pharmgkb.org/do/serve?objId=PA115&objCls=Gene) | CHRNB2 | PMID: [12177686](http://www.ncbi.nlm.nih.gov/pubmed/12177686) |
|  | [PA55](http://www.pharmgkb.org/do/serve?objId=PA55&objCls=Gene) | APOE | PMID: [20644562](http://www.ncbi.nlm.nih.gov/pubmed/20644562) |
|  | [PA128](http://www.pharmgkb.org/do/serve?objId=PA128&objCls=Gene) | CYP2D6 | PMID: [14674789](http://www.ncbi.nlm.nih.gov/pubmed/14674789) [11927765](http://www.ncbi.nlm.nih.gov/pubmed/11927765) [14674789](http://www.ncbi.nlm.nih.gov/pubmed/14674789) [12177686](http://www.ncbi.nlm.nih.gov/pubmed/12177686) |
|  | [PA130](http://www.pharmgkb.org/do/serve?objId=PA130&objCls=Gene) | CYP3A4 | PMID: [14674789](http://www.ncbi.nlm.nih.gov/pubmed/14674789) [11927765](http://www.ncbi.nlm.nih.gov/pubmed/11927765) [14674789](http://www.ncbi.nlm.nih.gov/pubmed/14674789) [12177686](http://www.ncbi.nlm.nih.gov/pubmed/12177686) |

Table S15. The gene information for “memantine”

| **ID** | **PharmGKB Accession Id** | **Gene Symbol** | **Publications** |
| --- | --- | --- | --- |
| No Gene data is found. | | | |

Table S16. The gene information for “rosiglitazone”

| **ID** | **PharmGKB Accession Id** | **Gene Symbol** | **Publications** |
| --- | --- | --- | --- |
|  | [PA134933118](http://www.pharmgkb.org/do/serve?objId=PA134933118&objCls=Gene) | ADIPOQ | PMID: [19553931](http://www.ncbi.nlm.nih.gov/pubmed/19553931) [18036314](http://www.ncbi.nlm.nih.gov/pubmed/18036314) |
|  | [PA29198](http://www.pharmgkb.org/do/serve?objId=PA29198&objCls=Gene) | HBA1 | PMID: [19553931](http://www.ncbi.nlm.nih.gov/pubmed/19553931) |
|  | [PA378](http://www.pharmgkb.org/do/serve?objId=PA378&objCls=Gene) | NR1I2 | PMID: [18547065](http://www.ncbi.nlm.nih.gov/pubmed/18547065) |
|  | [PA126](http://www.pharmgkb.org/do/serve?objId=PA126&objCls=Gene) | CYP2C9 | PMID: [18695978](http://www.ncbi.nlm.nih.gov/pubmed/18695978) |
|  | [PA134865839](http://www.pharmgkb.org/do/serve?objId=PA134865839&objCls=Gene) | SLCO1B1 | PMID: [19129086](http://www.ncbi.nlm.nih.gov/pubmed/19129086)[14977862](http://www.ncbi.nlm.nih.gov/pubmed/14977862)[17635496](http://www.ncbi.nlm.nih.gov/pubmed/17635496) |
|  | [PA29476](http://www.pharmgkb.org/do/serve?objId=PA29476&objCls=Gene) | HSD11B1 | PMID: none |
|  | [PA232](http://www.pharmgkb.org/do/serve?objId=PA232&objCls=Gene) | LPL | PMID: none |
|  | [PA181](http://www.pharmgkb.org/do/serve?objId=PA181&objCls=Gene) | NR3C1 | PMID: none |
|  | [PA281](http://www.pharmgkb.org/do/serve?objId=PA281&objCls=Gene) | PPARG | PMID: none |
|  | [PA422](http://www.pharmgkb.org/do/serve?objId=PA422&objCls=Gene) | RETN | PMID: none |
|  | [PA34890](http://www.pharmgkb.org/do/serve?objId=PA34890&objCls=Gene) | RXRA | PMID: none |
|  | [PA435](http://www.pharmgkb.org/do/serve?objId=PA435&objCls=Gene) | TNF | PMID: none |
|  | [PA30436](http://www.pharmgkb.org/do/serve?objId=PA30436&objCls=Gene) | LPIN1 | PMID: [18693052](http://www.ncbi.nlm.nih.gov/pubmed/18693052) |
|  | [PA125](http://www.pharmgkb.org/do/serve?objId=PA125&objCls=Gene) | CYP2C8 | PMID: [18303964](http://www.ncbi.nlm.nih.gov/pubmed/18303964) [17178266](http://www.ncbi.nlm.nih.gov/pubmed/17178266) [19129086](http://www.ncbi.nlm.nih.gov/pubmed/19129086) [16856883](http://www.ncbi.nlm.nih.gov/pubmed/16856883) [18695978](http://www.ncbi.nlm.nih.gov/pubmed/18695978) [19761371](http://www.ncbi.nlm.nih.gov/pubmed/19761371) |
|  | [PA55](http://www.pharmgkb.org/do/serve?objId=PA55&objCls=Gene) | APOE | PMID: [16770341](http://www.ncbi.nlm.nih.gov/pubmed/16770341) |
|  | [PA162396933](http://www.pharmgkb.org/do/serve?objId=PA162396933&objCls=Gene) | NAMPT | PMID: [17235334](http://www.ncbi.nlm.nih.gov/pubmed/17235334) |
|  | [PA34289](http://www.pharmgkb.org/do/serve?objId=PA34289&objCls=Gene) | RBP4 | PMID: [17235334](http://www.ncbi.nlm.nih.gov/pubmed/17235334) |
|  | [PA34921](http://www.pharmgkb.org/do/serve?objId=PA34921&objCls=Gene) | SAA1 | PMID: [16737350](http://www.ncbi.nlm.nih.gov/pubmed/16737350) |
|  | [PA34922](http://www.pharmgkb.org/do/serve?objId=PA34922&objCls=Gene) | SAA2 | PMID: [16737350](http://www.ncbi.nlm.nih.gov/pubmed/16737350) |
|  | [PA29009](http://www.pharmgkb.org/do/serve?objId=PA29009&objCls=Gene) | GSK3B | PMID: [19201691](http://www.ncbi.nlm.nih.gov/pubmed/19201691) |
|  | [PA30889](http://www.pharmgkb.org/do/serve?objId=PA30889&objCls=Gene) | MMP9 | PMID: [19201691](http://www.ncbi.nlm.nih.gov/pubmed/19201691) |
|  | [PA24684](http://www.pharmgkb.org/do/serve?objId=PA24684&objCls=Gene) | AKT1 | PMID: [16505118](http://www.ncbi.nlm.nih.gov/pubmed/16505118) [15788448](http://www.ncbi.nlm.nih.gov/pubmed/15788448) |
|  | [PA33942](http://www.pharmgkb.org/do/serve?objId=PA33942&objCls=Gene) | PTEN | PMID: [16505118](http://www.ncbi.nlm.nih.gov/pubmed/16505118) [15788448](http://www.ncbi.nlm.nih.gov/pubmed/15788448) |
|  | [PA28360](http://www.pharmgkb.org/do/serve?objId=PA28360&objCls=Gene) | FRAP1 | PMID: [16505118](http://www.ncbi.nlm.nih.gov/pubmed/16505118) |
|  | [PA33744](http://www.pharmgkb.org/do/serve?objId=PA33744&objCls=Gene) | PRKAA1 | PMID: [16505118](http://www.ncbi.nlm.nih.gov/pubmed/16505118) [17003345](http://www.ncbi.nlm.nih.gov/pubmed/17003345) |
|  | [PA33745](http://www.pharmgkb.org/do/serve?objId=PA33745&objCls=Gene) | PRKAA2 | PMID: [16505118](http://www.ncbi.nlm.nih.gov/pubmed/16505118) [17003345](http://www.ncbi.nlm.nih.gov/pubmed/17003345) |
|  | [PA34851](http://www.pharmgkb.org/do/serve?objId=PA34851&objCls=Gene) | RPS6KB1 | PMID: [16505118](http://www.ncbi.nlm.nih.gov/pubmed/16505118) |
|  | [PA33746](http://www.pharmgkb.org/do/serve?objId=PA33746&objCls=Gene) | PRKAB1 | PMID: [17003345](http://www.ncbi.nlm.nih.gov/pubmed/17003345) |
|  | [PA33747](http://www.pharmgkb.org/do/serve?objId=PA33747&objCls=Gene) | PRKAB2 | PMID: [17003345](http://www.ncbi.nlm.nih.gov/pubmed/17003345) |
|  | [PA33751](http://www.pharmgkb.org/do/serve?objId=PA33751&objCls=Gene) | PRKAG1 | PMID: [17003345](http://www.ncbi.nlm.nih.gov/pubmed/17003345) |
|  | [PA33752](http://www.pharmgkb.org/do/serve?objId=PA33752&objCls=Gene) | PRKAG2 | PMID: [17003345](http://www.ncbi.nlm.nih.gov/pubmed/17003345) |
|  | [PA24373](http://www.pharmgkb.org/do/serve?objId=PA24373&objCls=Gene) | ABCA1 | PMID: [18215356](http://www.ncbi.nlm.nih.gov/pubmed/18215356) |
|  | [PA33409](http://www.pharmgkb.org/do/serve?objId=PA33409&objCls=Gene) | PLIN | PMID: [16732015](http://www.ncbi.nlm.nih.gov/pubmed/16732015) |
|  | [PA267](http://www.pharmgkb.org/do/serve?objId=PA267&objCls=Gene) | ABCB1 | PMID: [20350646](http://www.ncbi.nlm.nih.gov/pubmed/20350646) |

Table S17. The gene information for “acetylcholine”

| **ID** | **PharmGKB Accession Id** | **Gene Symbol** | **Publications** |
| --- | --- | --- | --- |
|  | [PA114](http://www.pharmgkb.org/do/serve?objId=PA114&objCls=Gene) | CHRNA7 | PMID: [12036180](http://www.ncbi.nlm.nih.gov/pubmed/12036180) [15231719](http://www.ncbi.nlm.nih.gov/pubmed/15231719) [15695160](http://www.ncbi.nlm.nih.gov/pubmed/15695160) |
|  | [PA326](http://www.pharmgkb.org/do/serve?objId=PA326&objCls=Gene) | SLC18A3 | PMID: [15485505](http://www.ncbi.nlm.nih.gov/pubmed/15485505) |
|  | [PA113](http://www.pharmgkb.org/do/serve?objId=PA113&objCls=Gene) | CHRNA3 | PMID: [12036180](http://www.ncbi.nlm.nih.gov/pubmed/12036180) |
|  | [PA26496](http://www.pharmgkb.org/do/serve?objId=PA26496&objCls=Gene) | CHRNB4 | PMID: [12036180](http://www.ncbi.nlm.nih.gov/pubmed/12036180) |
|  | [PA20](http://www.pharmgkb.org/do/serve?objId=PA20&objCls=Gene) | ACHE | PMID: [15322258](http://www.ncbi.nlm.nih.gov/pubmed/15322258) |
|  | [PA26448](http://www.pharmgkb.org/do/serve?objId=PA26448&objCls=Gene) | CHAT | PMID: [12929139](http://www.ncbi.nlm.nih.gov/pubmed/12929139) |
|  | [PA435](http://www.pharmgkb.org/do/serve?objId=PA435&objCls=Gene) | TNF | PMID: [17507924](http://www.ncbi.nlm.nih.gov/pubmed/17507924) |
|  | [PA254](http://www.pharmgkb.org/do/serve?objId=PA254&objCls=Gene) | NOS3 | PMID: [14583681](http://www.ncbi.nlm.nih.gov/pubmed/14583681) [11740345](http://www.ncbi.nlm.nih.gov/pubmed/11740345) |

Table S18. The gene information for “nicotine”

| **ID** | **PharmGKB Accession Id** | **Gene Symbol** | **Publications** |
| --- | --- | --- | --- |
|  | [PA420](http://www.pharmgkb.org/do/serve?objId=PA420&objCls=Gene) | UGT1A1 | PMID: [12433823](http://www.ncbi.nlm.nih.gov/pubmed/12433823) |
|  | [PA37179](http://www.pharmgkb.org/do/serve?objId=PA37179&objCls=Gene) | UGT1A4 | PMID: [17576790](http://www.ncbi.nlm.nih.gov/pubmed/17576790) [12433823](http://www.ncbi.nlm.nih.gov/pubmed/12433823) |
|  | [PA419](http://www.pharmgkb.org/do/serve?objId=PA419&objCls=Gene) | UGT1A9 | PMID: [15470160](http://www.ncbi.nlm.nih.gov/pubmed/15470160) [12433823](http://www.ncbi.nlm.nih.gov/pubmed/12433823) |
|  | [PA37174](http://www.pharmgkb.org/do/serve?objId=PA37174&objCls=Gene) | UGT1A10 | PMID: [12433823](http://www.ncbi.nlm.nih.gov/pubmed/12433823) |
|  | [PA37178](http://www.pharmgkb.org/do/serve?objId=PA37178&objCls=Gene) | UGT1A3 | PMID: [12433823](http://www.ncbi.nlm.nih.gov/pubmed/12433823) |
|  | [PA37181](http://www.pharmgkb.org/do/serve?objId=PA37181&objCls=Gene) | UGT1A6 | PMID: [12433823](http://www.ncbi.nlm.nih.gov/pubmed/12433823) |
|  | [PA37182](http://www.pharmgkb.org/do/serve?objId=PA37182&objCls=Gene) | UGT1A7 | PMID: [12433823](http://www.ncbi.nlm.nih.gov/pubmed/12433823) |
|  | [PA37183](http://www.pharmgkb.org/do/serve?objId=PA37183&objCls=Gene) | UGT1A8 | PMID: [12433823](http://www.ncbi.nlm.nih.gov/pubmed/12433823) |
|  | [PA37188](http://www.pharmgkb.org/do/serve?objId=PA37188&objCls=Gene) | UGT2B15 | PMID: [12433823](http://www.ncbi.nlm.nih.gov/pubmed/12433823) |
|  | [PA361](http://www.pharmgkb.org/do/serve?objId=PA361&objCls=Gene) | UGT2B7 | PMID: [17576790](http://www.ncbi.nlm.nih.gov/pubmed/17576790) [15470160](http://www.ncbi.nlm.nih.gov/pubmed/15470160) [12433823](http://www.ncbi.nlm.nih.gov/pubmed/12433823) |
|  | [PA136](http://www.pharmgkb.org/do/serve?objId=PA136&objCls=Gene) | DBH | PMID: [16272956](http://www.ncbi.nlm.nih.gov/pubmed/16272956) |
|  | [PA193](http://www.pharmgkb.org/do/serve?objId=PA193&objCls=Gene) | HTR2A | PMID: [16272956](http://www.ncbi.nlm.nih.gov/pubmed/16272956) |
|  | [PA236](http://www.pharmgkb.org/do/serve?objId=PA236&objCls=Gene) | MAOA | PMID: [16272956](http://www.ncbi.nlm.nih.gov/pubmed/16272956) |
|  | [PA166](http://www.pharmgkb.org/do/serve?objId=PA166&objCls=Gene) | FMO3 | PMID: [1446003](http://www.ncbi.nlm.nih.gov/pubmed/1446003) |
|  | [PA37186](http://www.pharmgkb.org/do/serve?objId=PA37186&objCls=Gene) | UGT2B10 | PMID: [17576790](http://www.ncbi.nlm.nih.gov/pubmed/17576790) [18300939](http://www.ncbi.nlm.nih.gov/pubmed/18300939) [17909004](http://www.ncbi.nlm.nih.gov/pubmed/17909004) |
|  | [PA27092](http://www.pharmgkb.org/do/serve?objId=PA27092&objCls=Gene) | CYP1A1 | PMID: [10350185](http://www.ncbi.nlm.nih.gov/pubmed/10350185) |
|  | [PA27093](http://www.pharmgkb.org/do/serve?objId=PA27093&objCls=Gene) | CYP1A2 | PMID: [10350185](http://www.ncbi.nlm.nih.gov/pubmed/10350185) |
|  | [PA125](http://www.pharmgkb.org/do/serve?objId=PA125&objCls=Gene) | CYP2C8 | PMID: [10350185](http://www.ncbi.nlm.nih.gov/pubmed/10350185) |
|  | [PA126](http://www.pharmgkb.org/do/serve?objId=PA126&objCls=Gene) | CYP2C9 | PMID: [10350185](http://www.ncbi.nlm.nih.gov/pubmed/10350185) |
|  | [PA130](http://www.pharmgkb.org/do/serve?objId=PA130&objCls=Gene) | CYP3A4 | PMID: [10350185](http://www.ncbi.nlm.nih.gov/pubmed/10350185) |
|  | [PA31786](http://www.pharmgkb.org/do/serve?objId=PA31786&objCls=Gene) | NRXN1 | PMID: [18270208](http://www.ncbi.nlm.nih.gov/pubmed/18270208) |
|  | [PA119](http://www.pharmgkb.org/do/serve?objId=PA119&objCls=Gene) | CRH | PMID: [20554984](http://www.ncbi.nlm.nih.gov/pubmed/20554984) |
|  | [PA26874](http://www.pharmgkb.org/do/serve?objId=PA26874&objCls=Gene) | CRHR1 | PMID: [20554984](http://www.ncbi.nlm.nih.gov/pubmed/20554984) |
|  | [PA27010](http://www.pharmgkb.org/do/serve?objId=PA27010&objCls=Gene) | CTNNA3 | PMID: none |
|  | [PA26660](http://www.pharmgkb.org/do/serve?objId=PA26660&objCls=Gene) | CNGA3 | PMID: none |
|  | [PA28961](http://www.pharmgkb.org/do/serve?objId=PA28961&objCls=Gene) | GRB14 | PMID: none |
|  | [PA38800](http://www.pharmgkb.org/do/serve?objId=PA38800&objCls=Gene) | MYRIP | PMID: none |
|  | [PA38520](http://www.pharmgkb.org/do/serve?objId=PA38520&objCls=Gene) | PPP4R2 | PMID: none |
|  | [PA134955162](http://www.pharmgkb.org/do/serve?objId=PA134955162&objCls=Gene) | IBRDC1 | PMID: none |
|  | [PA28997](http://www.pharmgkb.org/do/serve?objId=PA28997&objCls=Gene) | GRM8 | PMID: none |
|  | [PA349](http://www.pharmgkb.org/do/serve?objId=PA349&objCls=Gene) | TBXAS1 | PMID: none |
|  | [PA31182](http://www.pharmgkb.org/do/serve?objId=PA31182&objCls=Gene) | MSRA | PMID: none |
|  | [PA30094](http://www.pharmgkb.org/do/serve?objId=PA30094&objCls=Gene) | KHDRBS3 | PMID: none |
|  | [PA26296](http://www.pharmgkb.org/do/serve?objId=PA26296&objCls=Gene) | CDH23 | PMID: none |
|  | [PA142671453](http://www.pharmgkb.org/do/serve?objId=PA142671453&objCls=Gene) | MICAL2 | PMID: none |
|  | [PA30725](http://www.pharmgkb.org/do/serve?objId=PA30725&objCls=Gene) | ME3 | PMID: none |
|  | [PA31930](http://www.pharmgkb.org/do/serve?objId=PA31930&objCls=Gene) | OPCML | PMID: none |
|  | [PA28980](http://www.pharmgkb.org/do/serve?objId=PA28980&objCls=Gene) | GRIN2B | PMID: none |
|  | [PA38116](http://www.pharmgkb.org/do/serve?objId=PA38116&objCls=Gene) | TRIM9 | PMID: none |
|  | [PA35854](http://www.pharmgkb.org/do/serve?objId=PA35854&objCls=Gene) | SLC24A4 | PMID: none |
|  | [PA28979](http://www.pharmgkb.org/do/serve?objId=PA28979&objCls=Gene) | GRIN2A | PMID: none |
|  | [PA134979792](http://www.pharmgkb.org/do/serve?objId=PA134979792&objCls=Gene) | C18orf34 | PMID: none |
|  | [PA36578](http://www.pharmgkb.org/do/serve?objId=PA36578&objCls=Gene) | TMPRSS3 | PMID: none |
|  | [PA26383](http://www.pharmgkb.org/do/serve?objId=PA26383&objCls=Gene) | CECR2 | PMID: none |
|  | [PA134944695](http://www.pharmgkb.org/do/serve?objId=PA134944695&objCls=Gene) | AK3L1 | PMID: none |
|  | [PA134952004](http://www.pharmgkb.org/do/serve?objId=PA134952004&objCls=Gene) | AK3L2 | PMID: none |
|  | [PA37228](http://www.pharmgkb.org/do/serve?objId=PA37228&objCls=Gene) | USH2A | PMID: none |
|  | [PA142671697](http://www.pharmgkb.org/do/serve?objId=PA142671697&objCls=Gene) | HEATR1 | PMID: none |
|  | [PA36393](http://www.pharmgkb.org/do/serve?objId=PA36393&objCls=Gene) | TCF7L1 | PMID: none |
|  | [PA37067](http://www.pharmgkb.org/do/serve?objId=PA37067&objCls=Gene) | TTN | PMID: none |
|  | [PA164741600](http://www.pharmgkb.org/do/serve?objId=PA164741600&objCls=Gene) | GRIK2 | PMID: none |
|  | [PA365](http://www.pharmgkb.org/do/serve?objId=PA365&objCls=Gene) | UPP1 | PMID: none |
|  | [PA26947](http://www.pharmgkb.org/do/serve?objId=PA26947&objCls=Gene) | CSMD1 | PMID: none |
|  | [PA37317](http://www.pharmgkb.org/do/serve?objId=PA37317&objCls=Gene) | VLDLR | PMID: none |
|  | [PA31818](http://www.pharmgkb.org/do/serve?objId=PA31818&objCls=Gene) | NTRK2 | PMID: none |
|  | [PA134956714](http://www.pharmgkb.org/do/serve?objId=PA134956714&objCls=Gene) | LUZP2 | PMID: none |
|  | [PA134889143](http://www.pharmgkb.org/do/serve?objId=PA134889143&objCls=Gene) | DCP1B | PMID: none |
|  | [PA28126](http://www.pharmgkb.org/do/serve?objId=PA28126&objCls=Gene) | FGF9 | PMID: none |
|  | [PA142670721](http://www.pharmgkb.org/do/serve?objId=PA142670721&objCls=Gene) | TMTC4 | PMID: none |
|  | [PA134911294](http://www.pharmgkb.org/do/serve?objId=PA134911294&objCls=Gene) | NIPA2 | PMID: none |
|  | [PA38273](http://www.pharmgkb.org/do/serve?objId=PA38273&objCls=Gene) | TRPM7 | PMID: none |
|  | [PA27773](http://www.pharmgkb.org/do/serve?objId=PA27773&objCls=Gene) | EMR1 | PMID: none |
|  | [PA134887312](http://www.pharmgkb.org/do/serve?objId=PA134887312&objCls=Gene) | FAM19A5 | PMID: none |
|  | [PA27103](http://www.pharmgkb.org/do/serve?objId=PA27103&objCls=Gene) | CYP2A7P1 | PMID: none |
|  | [PA27480](http://www.pharmgkb.org/do/serve?objId=PA27480&objCls=Gene) | DRD4 | PMID: [16272956](http://www.ncbi.nlm.nih.gov/pubmed/16272956) [17387332](http://www.ncbi.nlm.nih.gov/pubmed/17387332) |
|  | [PA26491](http://www.pharmgkb.org/do/serve?objId=PA26491&objCls=Gene) | CHRNA5 | PMID: [18618000](http://www.ncbi.nlm.nih.gov/pubmed/18618000) [20643934](http://www.ncbi.nlm.nih.gov/pubmed/20643934) [18385739](http://www.ncbi.nlm.nih.gov/pubmed/18385739) [20700147](http://www.ncbi.nlm.nih.gov/pubmed/20700147) |
|  | [PA140](http://www.pharmgkb.org/do/serve?objId=PA140&objCls=Gene) | DDC | PMID: [17184203](http://www.ncbi.nlm.nih.gov/pubmed/17184203) [15879433](http://www.ncbi.nlm.nih.gov/pubmed/15879433) |
|  | [PA124](http://www.pharmgkb.org/do/serve?objId=PA124&objCls=Gene) | CYP2C19 | PMID: [16740190](http://www.ncbi.nlm.nih.gov/pubmed/16740190) [10350185](http://www.ncbi.nlm.nih.gov/pubmed/10350185) |
|  | [PA129](http://www.pharmgkb.org/do/serve?objId=PA129&objCls=Gene) | CYP2E1 | PMID: [20233178](http://www.ncbi.nlm.nih.gov/pubmed/20233178) [12777962](http://www.ncbi.nlm.nih.gov/pubmed/12777962) [10350185](http://www.ncbi.nlm.nih.gov/pubmed/10350185) |
|  | [PA27102](http://www.pharmgkb.org/do/serve?objId=PA27102&objCls=Gene) | CYP2A7 | PMID: [16636685](http://www.ncbi.nlm.nih.gov/pubmed/16636685) [20136358](http://www.ncbi.nlm.nih.gov/pubmed/20136358) |
|  | [PA31945](http://www.pharmgkb.org/do/serve?objId=PA31945&objCls=Gene) | OPRM1 | PMID: [17224913](http://www.ncbi.nlm.nih.gov/pubmed/17224913) [15007373](http://www.ncbi.nlm.nih.gov/pubmed/15007373) |
|  | [PA34](http://www.pharmgkb.org/do/serve?objId=PA34&objCls=Gene) | ADRA1A | PMID: [17224913](http://www.ncbi.nlm.nih.gov/pubmed/17224913) |
|  | [PA26489](http://www.pharmgkb.org/do/serve?objId=PA26489&objCls=Gene) | CHRNA2 | PMID: [17224913](http://www.ncbi.nlm.nih.gov/pubmed/17224913) |
|  | [PA312](http://www.pharmgkb.org/do/serve?objId=PA312&objCls=Gene) | SLC6A4 | PMID: [16702982](http://www.ncbi.nlm.nih.gov/pubmed/16702982) |
|  | [PA115](http://www.pharmgkb.org/do/serve?objId=PA115&objCls=Gene) | CHRNB2 | PMID: [18593715](http://www.ncbi.nlm.nih.gov/pubmed/18593715) |
|  | [PA83](http://www.pharmgkb.org/do/serve?objId=PA83&objCls=Gene) | CACNA1C | PMID: [15635597](http://www.ncbi.nlm.nih.gov/pubmed/15635597) |
|  | [PA84](http://www.pharmgkb.org/do/serve?objId=PA84&objCls=Gene) | CACNA1D | PMID: [15635597](http://www.ncbi.nlm.nih.gov/pubmed/15635597) |
|  | [PA27101](http://www.pharmgkb.org/do/serve?objId=PA27101&objCls=Gene) | CYP2A13 | PMID: [16359169](http://www.ncbi.nlm.nih.gov/pubmed/16359169) [15860657](http://www.ncbi.nlm.nih.gov/pubmed/15860657) [17428784](http://www.ncbi.nlm.nih.gov/pubmed/17428784) [12920161](http://www.ncbi.nlm.nih.gov/pubmed/12920161) [16188955](http://www.ncbi.nlm.nih.gov/pubmed/16188955) [15528319](http://www.ncbi.nlm.nih.gov/pubmed/15528319) [11016631](http://www.ncbi.nlm.nih.gov/pubmed/11016631) [17922361](http://www.ncbi.nlm.nih.gov/pubmed/17922361) [12130698](http://www.ncbi.nlm.nih.gov/pubmed/12130698) [16917071](http://www.ncbi.nlm.nih.gov/pubmed/16917071) [17671098](http://www.ncbi.nlm.nih.gov/pubmed/17671098) [15063809](http://www.ncbi.nlm.nih.gov/pubmed/15063809) [17717079](http://www.ncbi.nlm.nih.gov/pubmed/17717079) [14633739](http://www.ncbi.nlm.nih.gov/pubmed/14633739) |
|  | [PA26496](http://www.pharmgkb.org/do/serve?objId=PA26496&objCls=Gene) | CHRNB4 | PMID: [18618000](http://www.ncbi.nlm.nih.gov/pubmed/18618000) [12036180](http://www.ncbi.nlm.nih.gov/pubmed/12036180) [18385739](http://www.ncbi.nlm.nih.gov/pubmed/18385739) |
|  | [PA121](http://www.pharmgkb.org/do/serve?objId=PA121&objCls=Gene) | CYP2A6 | PMID: [15861035](http://www.ncbi.nlm.nih.gov/pubmed/15861035) [15860657](http://www.ncbi.nlm.nih.gov/pubmed/15860657) [16135656](http://www.ncbi.nlm.nih.gov/pubmed/16135656) [16952495](http://www.ncbi.nlm.nih.gov/pubmed/16952495) [16359169](http://www.ncbi.nlm.nih.gov/pubmed/16359169) [20136358](http://www.ncbi.nlm.nih.gov/pubmed/20136358) [17206524](http://www.ncbi.nlm.nih.gov/pubmed/17206524) [16188955](http://www.ncbi.nlm.nih.gov/pubmed/16188955) [16272956](http://www.ncbi.nlm.nih.gov/pubmed/16272956) [20336063](http://www.ncbi.nlm.nih.gov/pubmed/20336063) [16758265](http://www.ncbi.nlm.nih.gov/pubmed/16758265) [11016631](http://www.ncbi.nlm.nih.gov/pubmed/11016631) [19300303](http://www.ncbi.nlm.nih.gov/pubmed/19300303) [10999944](http://www.ncbi.nlm.nih.gov/pubmed/10999944) [11207029](http://www.ncbi.nlm.nih.gov/pubmed/11207029) [19702528](http://www.ncbi.nlm.nih.gov/pubmed/19702528) [18388868](http://www.ncbi.nlm.nih.gov/pubmed/18388868) [17671098](http://www.ncbi.nlm.nih.gov/pubmed/17671098) [19365400](http://www.ncbi.nlm.nih.gov/pubmed/19365400) [14633739](http://www.ncbi.nlm.nih.gov/pubmed/14633739) [16402086](http://www.ncbi.nlm.nih.gov/pubmed/16402086) [15592323](http://www.ncbi.nlm.nih.gov/pubmed/15592323) [20554984](http://www.ncbi.nlm.nih.gov/pubmed/20554984) [17112808](http://www.ncbi.nlm.nih.gov/pubmed/17112808) [20233178](http://www.ncbi.nlm.nih.gov/pubmed/20233178) [18004205](http://www.ncbi.nlm.nih.gov/pubmed/18004205) [15564629](http://www.ncbi.nlm.nih.gov/pubmed/15564629) [16636685](http://www.ncbi.nlm.nih.gov/pubmed/16636685) [17428784](http://www.ncbi.nlm.nih.gov/pubmed/17428784) [15940289](http://www.ncbi.nlm.nih.gov/pubmed/15940289) [10544257](http://www.ncbi.nlm.nih.gov/pubmed/10544257) [17015050](http://www.ncbi.nlm.nih.gov/pubmed/17015050) [15475735](http://www.ncbi.nlm.nih.gov/pubmed/15475735) [10350185](http://www.ncbi.nlm.nih.gov/pubmed/10350185) [15735609](http://www.ncbi.nlm.nih.gov/pubmed/15735609) [11180041](http://www.ncbi.nlm.nih.gov/pubmed/11180041) [8627511](http://www.ncbi.nlm.nih.gov/pubmed/8627511) [17112802](http://www.ncbi.nlm.nih.gov/pubmed/17112802) [17717079](http://www.ncbi.nlm.nih.gov/pubmed/17717079) [15861044](http://www.ncbi.nlm.nih.gov/pubmed/15861044) [8937855](http://www.ncbi.nlm.nih.gov/pubmed/8937855) [17522595](http://www.ncbi.nlm.nih.gov/pubmed/17522595) [19279561](http://www.ncbi.nlm.nih.gov/pubmed/19279561) |
|  | [PA123](http://www.pharmgkb.org/do/serve?objId=PA123&objCls=Gene) | CYP2B6 | PMID: [18004205](http://www.ncbi.nlm.nih.gov/pubmed/18004205) [16135656](http://www.ncbi.nlm.nih.gov/pubmed/16135656) [17015050](http://www.ncbi.nlm.nih.gov/pubmed/17015050) [10350185](http://www.ncbi.nlm.nih.gov/pubmed/10350185) |
|  | [PA28371](http://www.pharmgkb.org/do/serve?objId=PA28371&objCls=Gene) | FREQ | PMID: [16402081](http://www.ncbi.nlm.nih.gov/pubmed/16402081) |
|  | [PA24684](http://www.pharmgkb.org/do/serve?objId=PA24684&objCls=Gene) | AKT1 | PMID: [12511591](http://www.ncbi.nlm.nih.gov/pubmed/12511591) |
|  | [PA113](http://www.pharmgkb.org/do/serve?objId=PA113&objCls=Gene) | CHRNA3 | PMID: [12511591](http://www.ncbi.nlm.nih.gov/pubmed/12511591) [20712524](http://www.ncbi.nlm.nih.gov/pubmed/20712524) [18618000](http://www.ncbi.nlm.nih.gov/pubmed/18618000) [12036180](http://www.ncbi.nlm.nih.gov/pubmed/12036180) [18385739](http://www.ncbi.nlm.nih.gov/pubmed/18385739) [20643934](http://www.ncbi.nlm.nih.gov/pubmed/20643934) |
|  | [PA26490](http://www.pharmgkb.org/do/serve?objId=PA26490&objCls=Gene) | CHRNA4 | PMID: [12511591](http://www.ncbi.nlm.nih.gov/pubmed/12511591) [16283473](http://www.ncbi.nlm.nih.gov/pubmed/16283473) [19290018](http://www.ncbi.nlm.nih.gov/pubmed/19290018) |
|  | [PA27478](http://www.pharmgkb.org/do/serve?objId=PA27478&objCls=Gene) | DRD2 | PMID: [20712524](http://www.ncbi.nlm.nih.gov/pubmed/20712524) [17189962](http://www.ncbi.nlm.nih.gov/pubmed/17189962) |
|  | [PA117](http://www.pharmgkb.org/do/serve?objId=PA117&objCls=Gene) | COMT | PMID: [18192898](http://www.ncbi.nlm.nih.gov/pubmed/18192898) |
|  | [PA114](http://www.pharmgkb.org/do/serve?objId=PA114&objCls=Gene) | CHRNA7 | PMID: [12511591](http://www.ncbi.nlm.nih.gov/pubmed/12511591) [10082212](http://www.ncbi.nlm.nih.gov/pubmed/10082212) [12036180](http://www.ncbi.nlm.nih.gov/pubmed/12036180) [15695160](http://www.ncbi.nlm.nih.gov/pubmed/15695160) |

Table S19. The gene information for “nimesulide”

| **ID** | **PharmGKB Accession Id** | **Gene Symbol** | **Publications** |
| --- | --- | --- | --- |
|  | [PA25623](http://www.pharmgkb.org/do/serve?objId=PA25623&objCls=Gene) | C1QB | PMID: [11810182](http://www.ncbi.nlm.nih.gov/pubmed/11810182) |
|  | [PA33856](http://www.pharmgkb.org/do/serve?objId=PA33856&objCls=Gene) | PSEN2 | PMID: [16331303](http://www.ncbi.nlm.nih.gov/pubmed/16331303) |
|  | [PA293](http://www.pharmgkb.org/do/serve?objId=PA293&objCls=Gene) | PTGS2 | PMID: [20548327](http://www.ncbi.nlm.nih.gov/pubmed/20548327) |

Table S20. The gene information for “donepezil”

| **ID** | **PharmGKB Accession Id** | **Gene Symbol** | **Publications** |
| --- | --- | --- | --- |
|  | [PA27114](http://www.pharmgkb.org/do/serve?objId=PA27114&objCls=Gene) | CYP3A | PMID: [18695978](http://www.ncbi.nlm.nih.gov/pubmed/18695978) |
|  | [PA131](http://www.pharmgkb.org/do/serve?objId=PA131&objCls=Gene) | CYP3A5 | PMID: [18695978](http://www.ncbi.nlm.nih.gov/pubmed/18695978) |
|  | [PA128](http://www.pharmgkb.org/do/serve?objId=PA128&objCls=Gene) | CYP2D6 | PMID: [20859244](http://www.ncbi.nlm.nih.gov/pubmed/20859244) [11927765](http://www.ncbi.nlm.nih.gov/pubmed/11927765) [18695978](http://www.ncbi.nlm.nih.gov/pubmed/18695978) [19738170](http://www.ncbi.nlm.nih.gov/pubmed/19738170) |
|  | [PA130](http://www.pharmgkb.org/do/serve?objId=PA130&objCls=Gene) | CYP3A4 | PMID: [18695978](http://www.ncbi.nlm.nih.gov/pubmed/18695978) [11927765](http://www.ncbi.nlm.nih.gov/pubmed/11927765) |
|  | [PA312](http://www.pharmgkb.org/do/serve?objId=PA312&objCls=Gene) | SLC6A4 | PMID: [18621671](http://www.ncbi.nlm.nih.gov/pubmed/18621671) |
|  | [PA26448](http://www.pharmgkb.org/do/serve?objId=PA26448&objCls=Gene) | CHAT | PMID: [16424819](http://www.ncbi.nlm.nih.gov/pubmed/16424819) |
|  | [PA55](http://www.pharmgkb.org/do/serve?objId=PA55&objCls=Gene) | APOE | PMID: [20644562](http://www.ncbi.nlm.nih.gov/pubmed/20644562) [12142731](http://www.ncbi.nlm.nih.gov/pubmed/12142731) |
|  | [PA25294](http://www.pharmgkb.org/do/serve?objId=PA25294&objCls=Gene) | BCHE | PMID: [20644562](http://www.ncbi.nlm.nih.gov/pubmed/20644562) |
|  | [PA27093](http://www.pharmgkb.org/do/serve?objId=PA27093&objCls=Gene) | CYP1A2 | PMID: [11927765](http://www.ncbi.nlm.nih.gov/pubmed/11927765) |

Table S21. The gene information for “tacrine”

| **ID** | **PharmGKB Accession Id** | **Gene Symbol** | **Publications** |
| --- | --- | --- | --- |
|  | [PA27093](http://www.pharmgkb.org/do/serve?objId=PA27093&objCls=Gene) | CYP1A2 | PMID: [8565783](http://www.ncbi.nlm.nih.gov/pubmed/8565783) [11927765](http://www.ncbi.nlm.nih.gov/pubmed/11927765) |
|  | [PA55](http://www.pharmgkb.org/do/serve?objId=PA55&objCls=Gene) | APOE | PMID: [9521254](http://www.ncbi.nlm.nih.gov/pubmed/9521254) [9777427](http://www.ncbi.nlm.nih.gov/pubmed/9777427) |
|  | [PA182](http://www.pharmgkb.org/do/serve?objId=PA182&objCls=Gene) | GSTM1 | PMID: [10801254](http://www.ncbi.nlm.nih.gov/pubmed/10801254) |
|  | [PA183](http://www.pharmgkb.org/do/serve?objId=PA183&objCls=Gene) | GSTT1 | PMID: [10801254](http://www.ncbi.nlm.nih.gov/pubmed/10801254) |
|  | [PA268](http://www.pharmgkb.org/do/serve?objId=PA268&objCls=Gene) | ABCB4 | PMID: [18004213](http://www.ncbi.nlm.nih.gov/pubmed/18004213) |
|  | [PA198](http://www.pharmgkb.org/do/serve?objId=PA198&objCls=Gene) | IL6 | PMID: [20415545](http://www.ncbi.nlm.nih.gov/pubmed/20415545) |

Table S22. The gene information for “choline”

| **ID** | **PharmGKB Accession Id** | **Gene Symbol** | **Publications** |
| --- | --- | --- | --- |
|  | [PA35879](http://www.pharmgkb.org/do/serve?objId=PA35879&objCls=Gene) | SLC2A4 | PMID: none |
|  | [PA37042](http://www.pharmgkb.org/do/serve?objId=PA37042&objCls=Gene) | TSHR | PMID: none |
|  | [PA37268](http://www.pharmgkb.org/do/serve?objId=PA37268&objCls=Gene) | VAMP3 | PMID: none |
|  | [PA25294](http://www.pharmgkb.org/do/serve?objId=PA25294&objCls=Gene) | BCHE | PMID: none |
|  | [PA331](http://www.pharmgkb.org/do/serve?objId=PA331&objCls=Gene) | SLC22A2 | PMID: none |
|  | [PA29189](http://www.pharmgkb.org/do/serve?objId=PA29189&objCls=Gene) | KALRN | PMID: none |
|  | [PA33383](http://www.pharmgkb.org/do/serve?objId=PA33383&objCls=Gene) | PLAUR | PMID: none |
|  | [PA34101](http://www.pharmgkb.org/do/serve?objId=PA34101&objCls=Gene) | RAB11A | PMID: none |
|  | [PA134950896](http://www.pharmgkb.org/do/serve?objId=PA134950896&objCls=Gene) | RAB11FIP3 | PMID: none |
|  | [PA134865095](http://www.pharmgkb.org/do/serve?objId=PA134865095&objCls=Gene) | RHOA | PMID: none |
|  | [PA36514](http://www.pharmgkb.org/do/serve?objId=PA36514&objCls=Gene) | TIAM1 | PMID: none |
|  | [PA43](http://www.pharmgkb.org/do/serve?objId=PA43&objCls=Gene) | AGTR1 | PMID: none |
|  | [PA24684](http://www.pharmgkb.org/do/serve?objId=PA24684&objCls=Gene) | AKT1 | PMID: none |
|  | [PA24791](http://www.pharmgkb.org/do/serve?objId=PA24791&objCls=Gene) | ANGPT1 | PMID: none |
|  | [PA24792](http://www.pharmgkb.org/do/serve?objId=PA24792&objCls=Gene) | ANGPT2 | PMID: none |
|  | [PA24793](http://www.pharmgkb.org/do/serve?objId=PA24793&objCls=Gene) | ANGPT4 | PMID: none |
|  | [PA25389](http://www.pharmgkb.org/do/serve?objId=PA25389&objCls=Gene) | BMX | PMID: none |
|  | [PA104](http://www.pharmgkb.org/do/serve?objId=PA104&objCls=Gene) | CDKN1A | PMID: none |
|  | [PA26880](http://www.pharmgkb.org/do/serve?objId=PA26880&objCls=Gene) | CRK | PMID: none |
|  | [PA27457](http://www.pharmgkb.org/do/serve?objId=PA27457&objCls=Gene) | DOK2 | PMID: none |
|  | [PA27744](http://www.pharmgkb.org/do/serve?objId=PA27744&objCls=Gene) | ELF1 | PMID: none |
|  | [PA27745](http://www.pharmgkb.org/do/serve?objId=PA27745&objCls=Gene) | ELF2 | PMID: none |
|  | [PA27749](http://www.pharmgkb.org/do/serve?objId=PA27749&objCls=Gene) | ELK1 | PMID: none |
|  | [PA27902](http://www.pharmgkb.org/do/serve?objId=PA27902&objCls=Gene) | ETS1 | PMID: none |
|  | [PA157](http://www.pharmgkb.org/do/serve?objId=PA157&objCls=Gene) | F2 | PMID: none |
|  | [PA28098](http://www.pharmgkb.org/do/serve?objId=PA28098&objCls=Gene) | FES | PMID: none |
|  | [PA28115](http://www.pharmgkb.org/do/serve?objId=PA28115&objCls=Gene) | FGF2 | PMID: none |
|  | [PA28194](http://www.pharmgkb.org/do/serve?objId=PA28194&objCls=Gene) | FN1 | PMID: none |
|  | [PA162388858](http://www.pharmgkb.org/do/serve?objId=PA162388858&objCls=Gene) | FOXO1 | PMID: none |
|  | [PA28237](http://www.pharmgkb.org/do/serve?objId=PA28237&objCls=Gene) | FOXO1A | PMID: none |
|  | [PA28454](http://www.pharmgkb.org/do/serve?objId=PA28454&objCls=Gene) | FYN | PMID: none |
|  | [PA28961](http://www.pharmgkb.org/do/serve?objId=PA28961&objCls=Gene) | GRB14 | PMID: none |
|  | [PA28962](http://www.pharmgkb.org/do/serve?objId=PA28962&objCls=Gene) | GRB2 | PMID: none |
|  | [PA28963](http://www.pharmgkb.org/do/serve?objId=PA28963&objCls=Gene) | GRB7 | PMID: none |
|  | [PA29941](http://www.pharmgkb.org/do/serve?objId=PA29941&objCls=Gene) | ITGA5 | PMID: none |
|  | [PA29953](http://www.pharmgkb.org/do/serve?objId=PA29953&objCls=Gene) | ITGB1 | PMID: none |
|  | [PA30621](http://www.pharmgkb.org/do/serve?objId=PA30621&objCls=Gene) | MAPK14 | PMID: none |
|  | [PA283](http://www.pharmgkb.org/do/serve?objId=PA283&objCls=Gene) | MAPK8 | PMID: none |
|  | [PA30877](http://www.pharmgkb.org/do/serve?objId=PA30877&objCls=Gene) | MMP2 | PMID: none |
|  | [PA31466](http://www.pharmgkb.org/do/serve?objId=PA31466&objCls=Gene) | NCK1 | PMID: none |
|  | [PA248](http://www.pharmgkb.org/do/serve?objId=PA248&objCls=Gene) | NFKB1 | PMID: none |
|  | [PA254](http://www.pharmgkb.org/do/serve?objId=PA254&objCls=Gene) | NOS3 | PMID: none |
|  | [PA32917](http://www.pharmgkb.org/do/serve?objId=PA32917&objCls=Gene) | PAK1 | PMID: none |
|  | [PA33308](http://www.pharmgkb.org/do/serve?objId=PA33308&objCls=Gene) | PIK3CA | PMID: none |
|  | [PA33312](http://www.pharmgkb.org/do/serve?objId=PA33312&objCls=Gene) | PIK3R1 | PMID: none |
|  | [PA33405](http://www.pharmgkb.org/do/serve?objId=PA33405&objCls=Gene) | PLG | PMID: none |
|  | [PA33955](http://www.pharmgkb.org/do/serve?objId=PA33955&objCls=Gene) | PTK2 | PMID: none |
|  | [PA33986](http://www.pharmgkb.org/do/serve?objId=PA33986&objCls=Gene) | PTPN11 | PMID: none |
|  | [PA34062](http://www.pharmgkb.org/do/serve?objId=PA34062&objCls=Gene) | PXN | PMID: none |
|  | [PA34232](http://www.pharmgkb.org/do/serve?objId=PA34232&objCls=Gene) | RASA1 | PMID: none |
|  | [PA296](http://www.pharmgkb.org/do/serve?objId=PA296&objCls=Gene) | RELA | PMID: none |
|  | [PA34851](http://www.pharmgkb.org/do/serve?objId=PA34851&objCls=Gene) | RPS6KB1 | PMID: none |
|  | [PA35746](http://www.pharmgkb.org/do/serve?objId=PA35746&objCls=Gene) | SHC1 | PMID: none |
|  | [PA338](http://www.pharmgkb.org/do/serve?objId=PA338&objCls=Gene) | STAT5A | PMID: none |
|  | [PA36441](http://www.pharmgkb.org/do/serve?objId=PA36441&objCls=Gene) | TEK | PMID: none |
|  | [PA435](http://www.pharmgkb.org/do/serve?objId=PA435&objCls=Gene) | TNF | PMID: none |
|  | [PA24852](http://www.pharmgkb.org/do/serve?objId=PA24852&objCls=Gene) | AP2A1 | PMID: none |
|  | [PA24855](http://www.pharmgkb.org/do/serve?objId=PA24855&objCls=Gene) | AP2M1 | PMID: none |
|  | [PA164741246](http://www.pharmgkb.org/do/serve?objId=PA164741246&objCls=Gene) | ARFGAP1 | PMID: none |
|  | [PA134879398](http://www.pharmgkb.org/do/serve?objId=PA134879398&objCls=Gene) | ARFIP2 | PMID: none |
|  | [PA134904544](http://www.pharmgkb.org/do/serve?objId=PA134904544&objCls=Gene) | ARHGAP10 | PMID: none |
|  | [PA164716055](http://www.pharmgkb.org/do/serve?objId=PA164716055&objCls=Gene) | ASAP1 | PMID: none |
|  | [PA26220](http://www.pharmgkb.org/do/serve?objId=PA26220&objCls=Gene) | CD4 | PMID: none |
|  | [PA26616](http://www.pharmgkb.org/do/serve?objId=PA26616&objCls=Gene) | CLTA | PMID: none |
|  | [PA26617](http://www.pharmgkb.org/do/serve?objId=PA26617&objCls=Gene) | CLTB | PMID: none |
|  | [PA26745](http://www.pharmgkb.org/do/serve?objId=PA26745&objCls=Gene) | COP | PMID: none |
|  | [PA26746](http://www.pharmgkb.org/do/serve?objId=PA26746&objCls=Gene) | COPA | PMID: none |
|  | [PA164718559](http://www.pharmgkb.org/do/serve?objId=PA164718559&objCls=Gene) | CYTH2 | PMID: none |
|  | [PA28595](http://www.pharmgkb.org/do/serve?objId=PA28595&objCls=Gene) | GBF1 | PMID: none |
|  | [PA28659](http://www.pharmgkb.org/do/serve?objId=PA28659&objCls=Gene) | GGA3 | PMID: none |
|  | [PA28816](http://www.pharmgkb.org/do/serve?objId=PA28816&objCls=Gene) | GOSR2 | PMID: none |
|  | [PA33849](http://www.pharmgkb.org/do/serve?objId=PA33849&objCls=Gene) | PSCD2 | PMID: none |
|  | [PA162408713](http://www.pharmgkb.org/do/serve?objId=PA162408713&objCls=Gene) | USO1 | PMID: none |
|  | [PA164714660](http://www.pharmgkb.org/do/serve?objId=PA164714660&objCls=Gene) | ACAP1 | PMID: none |
|  | [PA39](http://www.pharmgkb.org/do/serve?objId=PA39&objCls=Gene) | ADRB2 | PMID: none |
|  | [PA164716118](http://www.pharmgkb.org/do/serve?objId=PA164716118&objCls=Gene) | ASAP2 | PMID: none |
|  | [PA25189](http://www.pharmgkb.org/do/serve?objId=PA25189&objCls=Gene) | AVPR2 | PMID: none |
|  | [PA25355](http://www.pharmgkb.org/do/serve?objId=PA25355&objCls=Gene) | BIN1 | PMID: none |
|  | [PA26282](http://www.pharmgkb.org/do/serve?objId=PA26282&objCls=Gene) | CDH1 | PMID: none |
|  | [PA26406](http://www.pharmgkb.org/do/serve?objId=PA26406&objCls=Gene) | CENTB1 | PMID: none |
|  | [PA26618](http://www.pharmgkb.org/do/serve?objId=PA26618&objCls=Gene) | CLTC | PMID: none |
|  | [PA26824](http://www.pharmgkb.org/do/serve?objId=PA26824&objCls=Gene) | CPE | PMID: none |
|  | [PA27008](http://www.pharmgkb.org/do/serve?objId=PA27008&objCls=Gene) | CTNNA1 | PMID: none |
|  | [PA27013](http://www.pharmgkb.org/do/serve?objId=PA27013&objCls=Gene) | CTNNB1 | PMID: none |
|  | [PA27016](http://www.pharmgkb.org/do/serve?objId=PA27016&objCls=Gene) | CTNND1 | PMID: none |
|  | [PA27442](http://www.pharmgkb.org/do/serve?objId=PA27442&objCls=Gene) | DNM2 | PMID: none |
|  | [PA27618](http://www.pharmgkb.org/do/serve?objId=PA27618&objCls=Gene) | EDNRB | PMID: none |
|  | [PA134891660](http://www.pharmgkb.org/do/serve?objId=PA134891660&objCls=Gene) | EXOC1 | PMID: none |
|  | [PA134862170](http://www.pharmgkb.org/do/serve?objId=PA134862170&objCls=Gene) | EXOC2 | PMID: none |
|  | [PA134869816](http://www.pharmgkb.org/do/serve?objId=PA134869816&objCls=Gene) | EXOC3 | PMID: none |
|  | [PA134944654](http://www.pharmgkb.org/do/serve?objId=PA134944654&objCls=Gene) | EXOC4 | PMID: none |
|  | [PA35619](http://www.pharmgkb.org/do/serve?objId=PA35619&objCls=Gene) | EXOC5 | PMID: none |
|  | [PA134908462](http://www.pharmgkb.org/do/serve?objId=PA134908462&objCls=Gene) | EXOC6 | PMID: none |
|  | [PA134988420](http://www.pharmgkb.org/do/serve?objId=PA134988420&objCls=Gene) | EXOC7 | PMID: none |
|  | [PA29828](http://www.pharmgkb.org/do/serve?objId=PA29828&objCls=Gene) | IL2RA | PMID: none |
|  | [PA201](http://www.pharmgkb.org/do/serve?objId=PA201&objCls=Gene) | INS | PMID: none |
|  | [PA33329](http://www.pharmgkb.org/do/serve?objId=PA33329&objCls=Gene) | PIP5K1C | PMID: none |
|  | [PA34197](http://www.pharmgkb.org/do/serve?objId=PA34197&objCls=Gene) | RALA | PMID: none |
|  | [PA134892657](http://www.pharmgkb.org/do/serve?objId=PA134892657&objCls=Gene) | CEPT1 | PMID: none |
|  | [PA26468](http://www.pharmgkb.org/do/serve?objId=PA26468&objCls=Gene) | CHKA | PMID: none |
|  | [PA33099](http://www.pharmgkb.org/do/serve?objId=PA33099&objCls=Gene) | PCYT1A | PMID: none |
|  | [PA26448](http://www.pharmgkb.org/do/serve?objId=PA26448&objCls=Gene) | CHAT | PMID: none |
|  | [PA26826](http://www.pharmgkb.org/do/serve?objId=PA26826&objCls=Gene) | CPLX1 | PMID: none |
|  | [PA34132](http://www.pharmgkb.org/do/serve?objId=PA34132&objCls=Gene) | RAB3A | PMID: none |
|  | [PA38220](http://www.pharmgkb.org/do/serve?objId=PA38220&objCls=Gene) | RIMS1 | PMID: none |
|  | [PA326](http://www.pharmgkb.org/do/serve?objId=PA326&objCls=Gene) | SLC18A3 | PMID: none |
|  | [PA37838](http://www.pharmgkb.org/do/serve?objId=PA37838&objCls=Gene) | SLC5A7 | PMID: none |
|  | [PA35980](http://www.pharmgkb.org/do/serve?objId=PA35980&objCls=Gene) | SNAP25 | PMID: none |
|  | [PA36233](http://www.pharmgkb.org/do/serve?objId=PA36233&objCls=Gene) | STX1A | PMID: none |
|  | [PA36241](http://www.pharmgkb.org/do/serve?objId=PA36241&objCls=Gene) | STXBP1 | PMID: none |
|  | [PA36290](http://www.pharmgkb.org/do/serve?objId=PA36290&objCls=Gene) | SYT1 | PMID: none |
|  | [PA37267](http://www.pharmgkb.org/do/serve?objId=PA37267&objCls=Gene) | VAMP2 | PMID: none |
|  | [PA34977](http://www.pharmgkb.org/do/serve?objId=PA34977&objCls=Gene) | SCAMP2 | PMID: none |
|  | [PA114](http://www.pharmgkb.org/do/serve?objId=PA114&objCls=Gene) | CHRNA7 | PMID: [15231719](http://www.ncbi.nlm.nih.gov/pubmed/15231719) |
|  | [PA55](http://www.pharmgkb.org/do/serve?objId=PA55&objCls=Gene) | APOE | PMID: [8618881](http://www.ncbi.nlm.nih.gov/pubmed/8618881) |
|  | [PA24934](http://www.pharmgkb.org/do/serve?objId=PA24934&objCls=Gene) | ARF1 | PMID: none |
|  | [PA24942](http://www.pharmgkb.org/do/serve?objId=PA24942&objCls=Gene) | ARF6 | PMID: none |
|  | [PA30616](http://www.pharmgkb.org/do/serve?objId=PA30616&objCls=Gene) | MAPK1 | PMID: none |
|  | [PA249](http://www.pharmgkb.org/do/serve?objId=PA249&objCls=Gene) | NME1 | PMID: none |
|  | [PA33327](http://www.pharmgkb.org/do/serve?objId=PA33327&objCls=Gene) | PIP5K1A | PMID: none |
|  | [PA33397](http://www.pharmgkb.org/do/serve?objId=PA33397&objCls=Gene) | PLD2 | PMID: none |
|  | [PA34162](http://www.pharmgkb.org/do/serve?objId=PA34162&objCls=Gene) | RAC1 | PMID: none |

Table S23. The amount of genes in different drug class

| **ID** | **Drug Class** | **The amount of genes** |
| --- | --- | --- |
|  | Anticholinesterases | 6 |
|  | Ace Inhibitors, Plain | 24 |
|  | etanercept | 12 |
|  | rivastigmine | 2 |
|  | lithium | 13 |
|  | antiinflammatory and antirheumatic products, non-steroids | 11 |
|  | glatiramer acetate | 4 |
|  | hmg coa reductase inhibitors | 39 |
|  | curcumin | 2 |
|  | vitamin c | 16 |
|  | vitamin e | 1 |
|  | antidepressants | 43 |
|  | antipsychotics | 46 |
|  | galantamine | 7 |
|  | memantine | 0 |
|  | rosiglitazone | 34 |
|  | acetylcholine | 8 |
|  | nicotine | 88 |
|  | nimesulide | 2 |
|  | donepezil | 9 |
|  | tacrine | 6 |
|  | choline | 122 |
